# A chicken-origin *Ligilactobacillus agilis* R22 exerts probiotic features including growth-promotion and anti-Salmonella infection

**DOI:** 10.3389/fmicb.2026.1862425

**Published:** 2026-06-22

**Authors:** Yufan Sun, Xiaofen Zhang, Yaya Fan, Yi Liu, Delan Yang, Xiabing Chen, Lu Li

**Affiliations:** 1 National Key Laboratory of Agricultural Microbiology, College of Veterinary Medicine, Huazhong Agricultural University, Wuhan, China; 2 China Key Laboratory of Preventive Veterinary Medicine in Hubei Province, The Cooperative Innovation Center for Sustainable Pig Production, Wuhan, China; 3 Institute of Animal Husbandry and Veterinary Science, Wuhan Academy of Agricultural Sciences, Wuhan, China; 4 International Research Center for Animal Disease, Ministry of Science and Technology of the People's Republic of China, Wuhan, China

**Keywords:** chicken, growth performance, gut microbiota, *Ligilactobacillus agilis*, Salmonella

## Abstract

**Introduction:**

In the quest for the development of antibiotic alternatives, this study aimed to systematically evaluate the probiotic potential and mechanism of action of *Ligilactobacillus agilis* R22, a novel strain isolated from chickens.

**Methods:**

The *in vitro* probiotic properties of *L. agilis* R22, including growth ability, gastrointestinal tolerance, and antimicrobial activity, were initially assessed. Genomic and untargeted metabolomic analyses were conducted to explore its genetic and metabolic profiles. For *in vivo* evaluation, dietary supplementation of R22 was performed in broilers to assess growth performance, gut morphology, microbiota, and metabolites. Furthermore, a Salmonella enterica serovar Pullorum infection model in chicks and a fecal microbiota transplantation (FMT) experiment in mice were established to investigate its protective efficacy and underlying mechanisms.

**Results:**

*L. agilis* R22 exhibited robust growth, high tolerance to simulated gastrointestinal environments, and significant antimicrobial activity. Omics analyses revealed multiple probiotic-associated genes, an absence of virulence genes, and the capacity to synthesize essential nutrients for the host. In broilers, R22 supplementation significantly improved growth performance, intestinal morphology, and increased the levels of ghrelin and insulin-like growth factor-1. While the overall gut microbiota structure was not significantly altered, beneficial species and specific intestinal metabolites were enriched. In the S. Pullorum infection model, R22 effectively inhibited pathogen colonization in the gut and systemic dissemination to other organs, significantly mitigating pathological changes. Additionally, the FMT mouse model provided evidence that the *in vivo* protective effects of R22 are mediated by the gut microbiota it remodels.

**Discussion:**

The affected intestinal metabolites and the remodeled gut microbiota collectively contribute to the *in vivo* beneficial effects of *L. agilis* R22. Taken together, *L. agilis* R22 is a promising probiotic candidate that can be utilized in microbial feed additives.

## 1 Introduction

The long-term overuse of antibiotics as antimicrobial growth promoters (AGPs) in intensive animal husbandry has resulted in the emergence of multidrug-resistant pathogens (Uddin et al., 2021), which constitutes a severe threat to global public health (Van Puyvelde et al., 2018). In response to this crisis, countries worldwide have progressively implemented policies to restrict or ban the use of AGPs, heralding a new era of antibiotic-free animal production (Grave et al., 2006). Consequently, the development of safe and efficacious antibiotic alternatives to safeguard the health and sustainability of the livestock industry has become an urgent need.

Among the numerous antibiotic alternative products, probiotics have garnered considerable attention for their environmentally friendly and safe profile (Hill et al., 2014). Their beneficial mechanisms are multifaceted and cannot be ascribed to a single pathway (Sanders et al., 2019). Firstly, through competitive exclusion, probiotics compete with pathogens for adhesion sites on the intestinal epithelium and compete for limited nutrients, thereby physically constraining pathogen colonization (Sarkar and Mandal, 2016). Secondly, they actively produce a diverse array of antimicrobial substances (Vieco-Saiz et al., 2019). These include organic acids, such as lactic and acetic acid, which create an acidic milieu hostile to the growth of pathogens by lowering the local intestinal pH value (Ahmed et al., 2014). Moreover, the undissociated forms of these acids can penetrate the cell membrane of pathogen and disrupt their internal homeostasis (Scicutella et al., 2021). Many strains also synthesize highly targeted and potent bacteriocins and other compounds like hydrogen peroxide (Corr et al., 2007; Pridmore et al., 2008; Bertin et al., 2017). Furthermore, probiotics play a pivotal role in enhancing the intestinal barrier function. They stimulate goblet cells to secrete mucins, thickening the protective mucus layer (Tsirtsikos et al., 2012), and upregulate the expression of tight junction proteins such as occludin, claudin-1, and ZO-1(Wang et al., 2018). This action decreases intestinal permeability and prevents the translocation of harmful substances like endotoxins. Finally, probiotics act as potent immunomodulators. Their cell wall components, including peptidoglycan and lipoteichoic acid, are recognized by pattern recognition receptors, such as toll-like receptors, on host intestinal epithelial and immune cells. This recognition initiates signaling cascades that promote the secretion of anti-inflammatory cytokines and enhance the mucosal immune response, thereby sculpting a balanced and healthy intestinal immune environment (Shen et al., 2018; Kim et al., 2022). Among the diverse groups of probiotics, lactic acid bacteria (LAB) are considered one of the most promising feed additives, owing to their status as natural commensals of the animal gut, their safety, and their robust adaptability to the gastrointestinal environment (Niu et al., 2015).

*Lactobacillus* is an important member of LAB. The efficacy of *Lactobacillus* supplementation in poultry production is well-documented. In terms of enhancing production performance, the review showed that dietary inclusion of *Lactobacillus* significantly improves the growth performance of broilers, manifested as increased average daily gain (ADG) and a reduced feed conversion ratio (FCR; Neveling and Dicks, 2021; Wang et al., 2025). This improvement is not only a consequence of enhanced gut health but is also attributed to the ability of some *Lactobacillus* strains to secrete exogenous digestive enzymes, such as amylases and proteases, which assist the host in more efficiently breaking down and utilizing dietary nutrients (Liao and Nyachoti, 2017). Regarding the maintenance of intestinal health, intervention with *Lactobacillus* can significantly optimize the intestinal morphology. This is characterized by an increase in villus length (VL) and a decrease in crypt depth (CD) in the duodenum and jejunum, thereby enlarging the surface area available for nutrient absorption (Fuentes et al., 2013; Zhang et al., 2024). Such morphological improvements form a crucial physiological basis for their growth-promoting effects. In the context of antagonizing enteric pathogens, *Lactobacillus* act as a critical biological barrier. In poultry farming, pathogens such as Salmonella, *Clostridium perfringens* and *E. coli* are responsible for substantial economic losses (Bae et al., 2020; Wu et al., 2021; Alizadeh et al., 2023). Through mechanisms such as acid production and competitive adhesion, *Lactobacillus* effectively inhibit the colonization and proliferation of these pathogens in sites like the cecum, consequently reducing the incidence and severity of enteric diseases (Neveling et al., 2020). Furthermore, in boosting host immunity, *Lactobacillus* supplementation not only fortifies the local mucosal immunity but also exerts a positive influence on systemic immunity. For instance, it has been shown to enhance the immune response to vaccinations against critical viral diseases like Newcastle disease and infections bursal disease, as evidenced by elevated antibody titers (Smialek et al., 2018; Elbaz et al., 2021).

This study conducted a comprehensive evaluation of a newly isolated chicken-origin strain R22 belonging to *Ligilactobacillus agilis*, a species that was classified under the genus *Lactobacillus* prior to its reclassification based on the phylogenomic study (Zheng et al., 2020). *L. agilis* is naturally present in livestock, poultry, and humans. Unlike most lactobacilli, it exhibits motility ability and possesses additional advantages such as persistent colonization and the ability to express heterologous proteins (Suzuki et al., 2023), indicating its potential as a feed probiotic and a live delivery vehicle (Vezina et al., 2021). However, studies on its functions and applications remain limited. The investigation started with an *in vitro* assessment of its probiotic properties, followed by a multi-omics approach combining genomics and untargeted metabolomics to elucidate its genetic and metabolic basis. The dual efficacy in promoting growth and providing anti-infective protection was then validated *in vivo*. Finally, a fecal microbiota transplantation (FMT) experiment was performed to confirm that the action is closely associated with the remodeling of the gut microbiota. This research provided scientific evidences for the application of R22 as a novel microbial feed additive and novel data for the usage and genetic background of the species *L. agilis*.

## 2 Materials and methods

### 2.1 Bacterial strains and culture conditions

The probiotic candidate, *L. agilis* R22, was originally isolated from fresh fecal samples of healthy, free-range Qingjiao Ma chickens from local farms in Wuhan, Hubei Province, China. Briefly, the samples were homogenized in sterile saline to create a 10% (w/v) suspension. The resulting supernatant was then spread-plated onto de Man, Rogosa, and Sharpe (MRS; Qingdao Hi-Tech Industrial Park Hope Bio-Technology Co., Ltd.; Qingdao, China) agar plates. The plates were incubated anaerobically at 37 °C for 48 h.

Single colonies exhibiting distinct morphologies were selected and purified by repeated streaking on MRS agar. The purified isolates were subsequently screened for their gastrointestinal tolerance and antimicrobial activity and a single strain demonstrating superior probiotic potential was selected for further investigation. This strain was identified as *Ligilactobacillus agilis* via 16S rRNA gene sequencing, and designated as R22.

### 2.2 Gastrointestinal tract tolerance *in vitro*

Artificial gastric juice was formulated by adjusting the pH of MRS broth to 2.5 with dilute HCl, autoclaving at 121 °C for 15 min, adding 1.0 g of pepsin, and sterilizing through a 0.22 μm filter after thorough mixing. Artificial intestinal fluid was prepared by dissolving 0.68 g of KH_2_PO4 in 50 mL of distilled water, adjusting the pH to 6.8 with 0.1 mol/L NaOH, adding 1.0 g of trypsin, and bringing the final volume to 100 mL, followed by filter sterilization. Additionally, a bile salt-MRS broth was prepared by adding 0.1% (w/v) bile salts and 0.2% (w/v) sodium thioglycolate to MRS broth, which was subsequently filter-sterilized. The *L. agilis* R22 culture at the mid-logarithmic phase was inoculated into three distinct treatment solutions at a ratio of 1:10 (v/v), respectively, and incubated anaerobically at 37 °C for 2 h. Samples taken prior to treatment (*N*_0_) and after 2 h of incubation (*N*_*t*_) were used for viable cell counting. The survival rate of the strain was calculated according to the following equation: (*N*_*t*_/*N*_0_) × 100%.

### 2.3 Antimicrobial activity determination

#### 2.3.1 Pathogen culture

The pathogenic indicator strains used in this study is *Salmonella enterica* serovar Pullorum C79-13, which were obtained from our laboratory collection. The strains were cultured aerobically at 37 °C in Tryptic Soy Broth or on Tryptic Soy Agar (TSA; Qingdao Hi-Tech Industrial Park Hope Bio-Technology Co., Ltd., China).

#### 2.3.2 Agar well-diffusion assay

To assess the antimicrobial activity, an overnight culture of *S*. Pullorum C79-13 was adjusted to a turbidity equivalent to 0.5 McFarland standard using a turbidimeter, followed by a 10-fold dilution with sterile saline to achieve a final concentration of 1 × 107 CFU/mL. Then, 100 μL of the suspension was evenly spread onto the surface of TSA plates using a sterile cotton swb. Wells were then punched in the agar plates, and 200 μL of *L. agilis* R22 culture or other test samples were added to the respective wells. *L. agilis* R22 is a facultative anaerobe. The plates were incubated aerobically at 37 °C for 18 h, after which the diameters of the inhibition zones were measured.

### 2.4 Growth and pH value determination

To determine the growth and pH value of the bacterial culture, an overnight culture of *L. agilis* R22 was inoculated (1%, v/v) into fresh MRS broth and incubated under anaerobic conditions at 37 °C. At predetermined time points, samples were collected to monitor three parameters. The optical density at 600 nm (OD600) was measured using a microplate reader (Multiskan GO, Thermo Fisher Scientific, Waltham, MA, USA). Bacterial cells count (CFU/mL) were determined by the standard plate count method on MRS agar. Simultaneously, the pH of the culture broth was monitored using a pH meter.

### 2.5 Morphological observation by transmission electron microscopy (TEM)

*L. agilis* R22 was cultured to the mid-logarithmic phase. The bacterial cells were harvested by centrifugation (3,000 × g, 5 min) and washed twice with sterile ultrapure water. The cell pellet was then resuspended in sterile water to an appropriate concentration. A 10 μL aliquot of the bacterial suspension was applied onto a glow-discharged, carbon-coated copper grid (230 mesh) and allowed to adsorb for 1 min. After removing the excess liquid with filter paper, the grid was negatively stained with a drop of 2% (w/v) phosphotungstic acid for 1 min. The excess stain was again wicked away with filter paper, and the grid was air-dried at room temperature. Finally, the cellular morphology was observed and images were captured using a Talos L120C transmission electron microscope (TEM; Thermo Fisher Scientific, USA) operating at an accelerating voltage of 120 kV.

### 2.6 Characterization of the antimicrobial substances

The pathogenic indicator strains used in this study were *S*. Pullorum C79-13, *Salmonella enterica* serovar Typhimurium ATCC 14028, and Extraintestinal pathogenic *Escherichia coli* PCN033, all of which were obtained from our laboratory collection.

To characterize the nature of the antimicrobial substance, cell-free supernatant (CFS) was prepared from an overnight culture of *L. agilis* R22 by centrifugation (10,000 × g, 1 min) followed by filtration through a 0.22 μm membrane filter (Merck KGaA, Darmstadt, Germany). The cell-free supernatant (CFS) was then divided into aliquots and subjected to the following treatments: (i) heat treatment (100 °C for 10 min); (ii) enzymatic digestion with Proteinase K (Beyotime Biotech Inc., China) at a final concentration of 1 mg/mL for 2 h at 37 °C; and (iii) pH neutralization by adjusting the pH to 6.0 with 1 M NaOH. Wells were created in the agar plates, and 200 μL of the *L. agilis* R22 whole culture, the untreated CFS, and each of the treated CFS samples were added to separate wells. Antimicrobial activity was determined following the exact same procedures as described in Section 2.3.

### 2.7 Antibiotic susceptibility tests

The Minimum Inhibitory Concentrations (MICs) of nine different antibiotics against *L. agilis* R22 were determined using the broth microdilution method. The assay was performed in strict accordance with the guidelines established by the European Food Safety Authority (EFSA) for assessing the antibiotic susceptibility of microbial feed additives (Rychen et al., 2018). The antibiotics tested included ampicillin, vancomycin, gentamicin, kanamycin, streptomycin, clindamycin, tetracycline, chloramphenicol, and erythromycin (Shanghai Yuanye Bio-Technology Co., Ltd., China). Following incubation of the 96-well-plates under anaerobic conditions at 37 °C for 18 h, the MIC was defined as the lowest concentration of an antibiotic that resulted in no visible bacterial growth.

### 2.8 Draft-genome sequencing, assembly, and bioinformatic analysis

#### 2.8.1 Draft-genome sequencing and assembly

Genomic DNA of *L. agilis* R22 was extracted using TIANamp Bacteria DNA Kit DP302 (TIANGEN Biotech, China). Following quantification and quality assessment, a paired-end sequencing library was constructed using the NEXTFLEX Rapid DNA-Seq Kit (Bioo Scientific Corp., USA). The library was then sequenced on an Illumina NextSeq 2000 platform (Illumina, Inc., USA), generating 2 × 150 bp reads. The raw sequencing data were quality-filtered using fastp (v0.20.0), and the resulting clean data were assembled into scaffolds using SOAP *de novo* (v2.04).

#### 2.8.2 Bioinformatic analysis

All of the analyses were performed using the Majorbio Cloud Platform (www.majorbio.com) from Shanghai Majorbio Bio-pharm Technology Co., Ltd (Shanghai, China). Gene prediction was conducted using Prodigal (v2.6.3), tRNAscan-SE (v2.0.12), and Barrnap (v0.9). The predicted coding sequences (CDSs) were functionally annotated by alignment against multiple databases, including the NCBI Non-Redundant Protein (NR), Swiss-Prot, Pfam, Gene Ontology (GO), Clusters of Orthologous Groups (COG), and Kyoto Encyclopedia of Genes and Genomes (KEGG) databases.

For safety assessment, potential antibiotic resistance genes (ARGs) and virulence factors were identified using ResFinder (v4.3.1), the Comprehensive Antibiotic Resistance Database (CARD, v3.2.6), PathogenFinder (v1.1), and the Virulence Factor Database (VFDB). Secondary metabolite gene clusters were predicted using antiSMASH (v6.1.1) and BAGEL4.

For taxonomic identification, a multilocus sequence analysis phylogenetic tree was constructed based on the concatenated sequences of 31 housekeeping genes from strain *L. agilis* R22 and 19 closely related reference strains. The tree was generated using the Neighbor-Joining method in MEGA (v6.0). Additionally, the Average Nucleotide Identity (ANI) between the strain R22 and the reference genomes was calculated using MUMmer (v3.23), with a threshold of >95% for species delineation. The genomic circular map was visualized using Circos (v0.69.6)

### 2.9 Untargeted metabolomics analysis of CFS of *L. agilis* R22

#### 2.9.1 Sample preparation

*L. agilis* R22 was cultured anaerobically in MRS broth at 37 °C. CFS samples were collected at the mid-logarithmic phase (6 h, designated as R22-Con) and the stationary phase (24 h, designated as R22). Uninoculated MRS broth served as the blank control. For metabolite extraction, 200 μL of each sample was mixed with 800 μL of a pre-chilled extraction solvent (methanol/acetonitrile, 1:1, v/v) containing an internal standard (0.02 mg/mL). The mixture was then subjected to low-temperature ultrasonication, followed by precipitation at −20 °C and centrifugation (13,000 × g, 15 min). The resulting supernatant was collected and dried under a gentle stream of nitrogen gas. The dried residue was reconstituted in 120 μL of a reconstitution solvent (acetonitrile/water, 1:1, v/v), centrifuged, and the final supernatant was transferred to an autosampler vial for LC-MS analysis. A quality control (QC) sample was prepared by pooling equal aliquots from all experimental samples.

#### 2.9.2 LC-MS analysis

The LC-MS/MS analysis of sample was conducted on a Thermo UHPLC-Q Exactive HF-X system equipped with an ACQUITY HSS T3 column (100 mm × 2.1 mm i.d., 1.8 μm; Waters, USA). The mobile phase consisted of solvent A (water/acetonitrile, 95:5, v/v, with 0.1% formic acid) and solvent B (acetonitrile/isopropanol/water, 47.5:47.5:5, v/v/v, with 0.1% formic acid). An injection volume of 3 μL was used for each sample. The mass spectrometer was operated with an electrospray ionization source in both positive and negative ion modes. Data were acquired over a scan range of m/z 70–1,050. Key ESI parameters were set as follows: sheath gas and auxiliary gas flow rates were 50 and 13 arb, respectively; the auxiliary gas heater temperature was 425 °C, and the capillary temperature was 325 °C. The spray voltage was set to +3.5 kV for positive mode and −3.5 kV for negative mode, with an S-Lens RF level of 50. For MS/MS fragmentation, a stepped Normalized Collision Energy (NCE) of 20%, 40%, and 60% was applied. The resolution was set to 60,000 for full MS scans and 7,500 for MS/MS scans. To ensure data quality, a QC sample, prepared by pooling equal aliquots from all samples, was injected every 5–15 experimental samples throughout the analytical run.

#### 2.9.3 Data processing

The raw LC-MS data were processed using Progenesis QI (v3.0, Waters Corp., USA) on the Majorbio Cloud Platform (www.majorbio.com) from Shanghai Majorbio Bio-pharm Technology Co., Ltd. (Shanghai, China). The workflow included baseline filtering, peak picking, peak alignment, and feature extraction. Metabolite identification was performed by matching the accurate mass and MS/MS fragmentation patterns of the features against public databases such as the Human Metabolome Database (HMDB) and METLIN, with a mass tolerance of less than 10 ppm. The resulting data matrix was subjected to a rigorous preprocessing pipeline. Features were first filtered to remove those with a relative standard deviation ≥30% in the QC samples. The data were then normalized by total sum normalization and subsequently log10-transformed for statistical analysis.

Multivariate statistical analysis was conducted to assess inter-group differences. Partial Least Squares Discriminant Analysis (PLS-DA) and Orthogonal PLS-DA (OPLS-DA) were employed to visualize sample clustering and identify group-specific metabolic patterns. The validity of these models was confirmed using a permutation test (e.g., *n* = 200 permutations). The statistical significance of the group separation was further evaluated by Analysis of Similarities (ANOSIM).

Differential metabolites were screened based on a dual criterion: a Variable Importance in Projection (VIP) value ≥ 1.0 from the OPLS-DA model and a *p* < 0.05. The identified differential metabolites were then subjected to KEGG pathway enrichment analysis. A hierarchical clustering heatmap was generated using the SciPy library (v1.0.0) in Python to visualize the expression patterns of these differential metabolites across the groups.

### 2.10 Effect of *L. agilis* R22 on the growth performance of broilers

#### 2.10.1 Animals, diets, and experimental design

A total of 320 1-day-old Arbor Acres broiler chicks with similar initial body weights were randomly allocated to two treatment groups, with 16 replicate cages per group and 10 chicks per cage. The feeding trial lasted for 40 days. The two treatment groups were as follows: (1) Con group, fed a basal diet; and (2) R22 group, fed the basal diet supplemented with 2 × 108 CFU/kg of lyophilized *L. agilis* R22 powder. All broilers were housed under standard management conditions with *ad libitum* access to feed and water. The basal diet was formulated to meet the nutritional requirements for chickens according to the Chinese Feeding Standard (NY/T 33-2004; Table 1). The feed was provided in crumble form from day 1 to 10 and in pellet form from day 11 to 40. No antibiotics or other growth promoters were used throughout the experimental period.

**Table 1:** Composition of basal diets.

Items	Day 1–21	Day 22–40
Ingredient, %		
Corn	49.05	36.57
Flour	9.99	15.00
Corn protein powder	2.00	3.00
Peanut meal	0.00	2.00
Cottonseed meal	4.00	3.00
Soybean meal	30.18	26.39
Chicken bone meal	1.00	2.70
Duck fat	0.00	8.20
Limestone powder	1.03	0.96
L-lysine sulfate	0.52	0.63
Methionine hydroxy analog	0.38	0.46
L-threonine	0.16	0.19
L-valine	0.11	0.13
Choline chloride	0.10	0.10
Calcium hydrogen phosphate	0.81	0.00
Sodium chloride	0.25	0.25
Phytase	0.02	0.02
Vitamin premix	0.25	0.25
Mineral trace elements	0.15	0.15
Total	100.00	100.00
Nutrient levels, %		
Dry matter	87.260	88.290
Crude protein	23.000	23.000
Crude fat	2.590	10.450
Crude fiber	3.110	2.790
Salt content	0.250	0.270
Calcium	0.800	0.750
Total phosphorus	0.600	0.500
Available phosphorus	0.300	0.250
Total lysine	1.446	1.444
Total methionine	0.691	0.749
Total tyrosine	1.017	1.017
Total tryptophan	0.262	0.250
Total arginine	1.514	1.492
Total valine	1.142	1.131
Total isoleucine	0.934	0.906
Total leucine	1.930	1.901
Metabolizable energy, kcal/kg	2,850	3,300

Vitamin premix provided the following per kg of complete diet: Vitamin A 12,000 IU, Vitamin D3 3,000 IU, Vitamin E 30 mg, Vitamin K3 3 mg, Thiamine (B1) 3 mg, Riboflavin (B_2_) 8 mg, Pyridoxine (B6) 4 mg, Vitamin B1_2_ 0.02 mg, Niacin 50 mg, Pantothenic acid 15 mg, Folic acid 1 mg, Biotin 0.2 mg, and Choline chloride 500 mg.

Mineral trace element premix provided the following per kg of complete diet: Zn 80 mg, Mn 100 mg, Fe 80 mg, Cu 8 mg, I 0.7 mg, and Se 0.3 mg.

#### 2.10.2 Growth performance measurement

On days 1, 14, 21, and 40 of the experiment, the body weight (BW) and feed intake were recorded on a per-replicate (cage) basis. These data were used to calculate the ADG, average daily feed intake (ADFI), and FCR.

#### 2.10.3 Sample collection and analysis

On day 40 of the experiment, one broiler was randomly selected from each of eight replicate cages per group for sample collection. The wing vein area was disinfected with 75% ethanol prior to blood collection by venipuncture using a syringe, after which digital pressure was applied for hemostasis. Blood samples were collected and centrifuged (3,000 × g, 15 min, 4 °C) to obtain serum. The serum concentrations of growth hormone (GH), ghrelin, and insulin-like growth factor-1 (IGF-1) were determined using commercial ELISA kits (HYCEZMBIO, China) according to the manufacturer's instructions. All experimental broilers were euthanized by cervical dislocation for sampling. Segments of the duodenum were collected for histological analysis. Contents from the duodenum and cecum were aseptically collected, immediately snap-frozen in liquid nitrogen, and stored at −80 °C for subsequent gut microbiota and untargeted metabolomics analyses. Contents from the other cecum were collected for the analysis of short-chain fatty acids (SCFAs).

#### 2.10.4 Intestinal morphological analysis

For histological examination, duodenum segments from six randomly selected broilers per group were fixed in 4% paraformaldehyde. The fixed tissues were then dehydrated through a graded ethanol series, embedded in paraffin, and sectioned into 4 μm-thick slices. The sections were stained with hematoxylin and eosin (H&E) and examined under a light microscope (Nikon Eclipse CI, Japan). For morphometric analysis, 10 well-oriented, intact villi were randomly selected from each of six sections per group. The VL and CD were measured using Image-Pro Plus 6.0 software (Media Cybernetics, Inc., USA), and the VL/CD ratio was calculated.

#### 2.10.5 Gut microbiota analysis

Total microbial genomic DNA was extracted from the duodenal and cecal contents using the TIANamp Stool DNA Kit (TIANGEN Biotech, China) according to the manufacturer's protocols. The V3–V4 hypervariable region of the 16S rRNA gene was amplified by PCR using the specific primer pair 338F 5′-ACTCCTACGGGAGGCAGCAG3′) and 806R 5′-GGACTACHVGGGTWTCTAAT3′). The resulting amplicons were then used for library construction and paired-end sequencing on an Illumina NextSeq 2000 platform by Majorbio Bio-pharm Technology Co., Ltd. (Shanghai, China). The raw sequencing data have been deposited in the NCBI Sequence Read Archive (SRA) database (accession number PRJNA1263642).

The raw sequence reads were quality-filtered and merged using fastp and FLASH, respectively. The merged sequences were then processed and denoised using the DADA2 pipeline to generate Amplicon Sequence Variants (ASVs). Taxonomic classification of the ASVs was performed using a Naive Bayes classifier against the SILVA database (v138.2/16s_bacteria). All of the analyses were performed using the Majorbio Cloud Platform (www.majorbio.com) from Shanghai Majorbio Bio-pharm Technology Co., Ltd. Alpha diversity (Chao1 richness and Shannon diversity indices) was calculated using Mothur (v1.30.2). Differences in alpha diversity between groups were evaluated using the Wilcoxon rank-sum test. Beta diversity was analyzed to assess the structural differences between microbial communities. Principal Coordinate Analysis (PCoA) and Non-metric Multidimensional Scaling (NMDS) were performed based on Bray-Curtis dissimilarity matrices and visualized using the vegan package (v2.5–3) in R. The statistical significance of the group clustering was tested by ANOSIM and Permutational Multivariate Analysis of Variance (PERMANOVA) with 999 permutations. Finally, Linear discriminant analysis Effect Size (LEfSe) was employed to identify differentially abundant taxa between the groups. LDA score >2.0 was used to define significant biomarkers.

#### 2.10.6 Untargeted metabolomics analysis of intestinal contents

Approximately 50 mg of duodenal and cecal content samples were accurately weighed and subjected to untargeted metabolomics analysis. Sample preparation, LC-MS detection, data processing, and statistical analysis were performed following the exact same procedures as described in Section 2.8.

#### 2.10.7 Quantification of cecal SCFA

Approximately 25 mg of cecal content was accurately weighed and homogenized in 500 μL of an aqueous solution containing 0.5% phosphoric acid and an internal standard (2-ethylbutyric acid, 10 μg/mL). The homogenization was performed using a high-throughput tissue grinder. The resulting homogenate was ultrasonicated in an ice bath for 10 min and then centrifuged at 13,000 × g for 15 min at 4 °C. For derivatization and extraction, 200 μL of the supernatant was mixed with 200 μL of n-butanol, vortexed, and ultrasonicated in an ice bath. After a final centrifugation step (13,000 × g, 5 min, 4 °C), the upper n-butanol phase containing the derivatized SCFAs was transferred to an autosampler vial for GC-MS analysis. The analysis was conducted on an Agilent 8890B-7000D GC-MS system (Agilent Technologies, USA).

### 2.11 Protective effect of *L. agilis* R22 against *S*. Pullorum infection in chicks

#### 2.11.1 Animals, experimental design, and challenge model

A total of 54 one-day-old Specific-Pathogen-Free White Leghorn chicks, hatched from eggs sourced from Boehringer Ingelheim Vital Biotechnology (Beijing) Co., Ltd., were randomly assigned to three groups (*n* = 18 per group): (1) NC group, unchallenged and orally administered with sterile saline; (2) SP group, Challenged with *S*. Pullorum and orally administered with sterile saline; (3) R22 group, challenged with *S*. Pullorum and orally administered with strain R22.

From day 1 to day 9, chickens in the R22 group were orally daily with 1 × 108 CFU of the R22 bacterial suspension in a volume of 500 μL. Chickens in the NC and SP groups received an equal volume of sterile saline. On day 4, chickens in the SP and R22 groups were orally challenged with 1.2 × 10^9^ CFU of *S*. Pullorum C79-13 in a 500 μL volume.

#### 2.11.2 Sample collection and analysis

The BW of each chicken was recorded daily throughout the experiment. On day 9, all surviving chicks were euthanized by cervical dislocation for sampling. The samples of the liver and cecum were collected for histopathological analysis, which was performed as described in Section 2.9. For the quantification of pathogen load, six chickens per group were randomly selected and euthanized on days 4, 6, and 9. The liver, spleen, and cecum were aseptically collected. The tissues were accurately weighed and homogenized in nine volumes of sterile saline to create a 10% (w/v) tissue homogenate. The homogenate was serially diluted and plated onto TSA for enumeration of Salmonella colonies after incubation at 37 °C for 18 h.

### 2.12 Protective effect of FMT against *S*. Typhimurium infection in mice

#### 2.12.1 Preparation of donor mice and fecal slurry

A total of sixteen mice were randomly assigned to two donor groups (*n* = 8): a control donor group (Con-D) and an R22 donor group (R22-D). Mice in the R22-D group were orally every other day from day-6 to day 12 with 1.0 × 107 CFU of *L. agilis* R22 suspension (50 μL). Mice in the Con-D group received an equal volume of sterile saline.

To prepare the fecal slurry, fresh fecal pellets were collected from the donor mice at each time of administration. The pellets were immediately suspended in sterile saline at a ratio of 1:6 (w/v, feces to saline). The mixture was then thoroughly homogenized with a steel bead in a tissue grinder (60 Hz for 40 s). The resulting slurry was centrifuged at 500 × g for 3 min at 4 °C. Collect the supernatant and repeat the centrifugation step on the collected supernatant. The final supernatant was used as the fecal slurry for transplantation

#### 2.12.2 Experimental design and animal model

A total of 40 3-week-old mice were randomly assigned to five groups (*n* = 8): (1) NC group, unchallenged and received sterile saline; (2) ST group, challenged with *S*. Typhimurium and received sterile saline; (3) R22 (ST) group, pre-treated with *L. agilis* R22 and challenged with *S*. Typhimurium; (4) Con-FMT (ST) group, pre-treated with fecal slurry from control donors (Con-D) and challenged with *S*. Typhimurium; (5) R22-FMT (ST) group, pre-treated with fecal slurry from R22-treated donors (R22-D) and challenged with *S*. Typhimurium.

The experiment was conducted over a 14-day period. On days 2, 4, 6, 8, 10, and 12, mice in the R22 (ST) group were orally with 1.0 × 107 CFU of *L. agilis* R22. Mice in the Con-FMT (ST) and R22-FMT (ST) groups were orally with the corresponding fecal slurries from their respective donor groups. The orally volume for all treatments was 50 μL. On day 9, all mice, except for those in the NC group, were orally challenged with 2 × 107 CFU of *S*. Typhimurium ATCC 14028 in a 100 μL volume. Mice in the NC group received an equal volume of sterile saline.

#### 2.12.3 Sample collection and analysis

The BW of each mouse was recorded daily throughout the experiment. On day 6 post-infection (day 14 of the experiment), blood was collected from the retro-orbital venous plexus of all mice, followed by euthanasia via cervical dislocation. The cecum was collected for histopathological analysis and pathogen load quantification, following the same procedures as described previously. Blood samples were collected, and the resulting serum was used to measure the concentrations of the inflammatory cytokines IL-6, IL-12, IL-1β, and TNF-α using commercial ELISA kits (MultiSciences Biotech Co., Ltd., Hangzhou, China).

### 2.13 Statistical analysis

Data related to growth performance, body weight ratios, and SCFA concentrations were analyzed using a two-way ANOVA. Unless otherwise specified, all other data were analyzed using a two-tailed Student's *t*-test. All statistical analyses were performed using GraphPad Prism 9.5.0. Data are presented as the mean ± standard deviation (SD). Statistical significance was defined at different levels: *p* < 0.05, ^*^p < 0.05, ^**^p < 0.00, ^***^p < 0.001 and ^****^p < 0.0001.

## 3 Results

### 3.1 Biological characteristics of *L. agilis* R22

**Figure 1:**
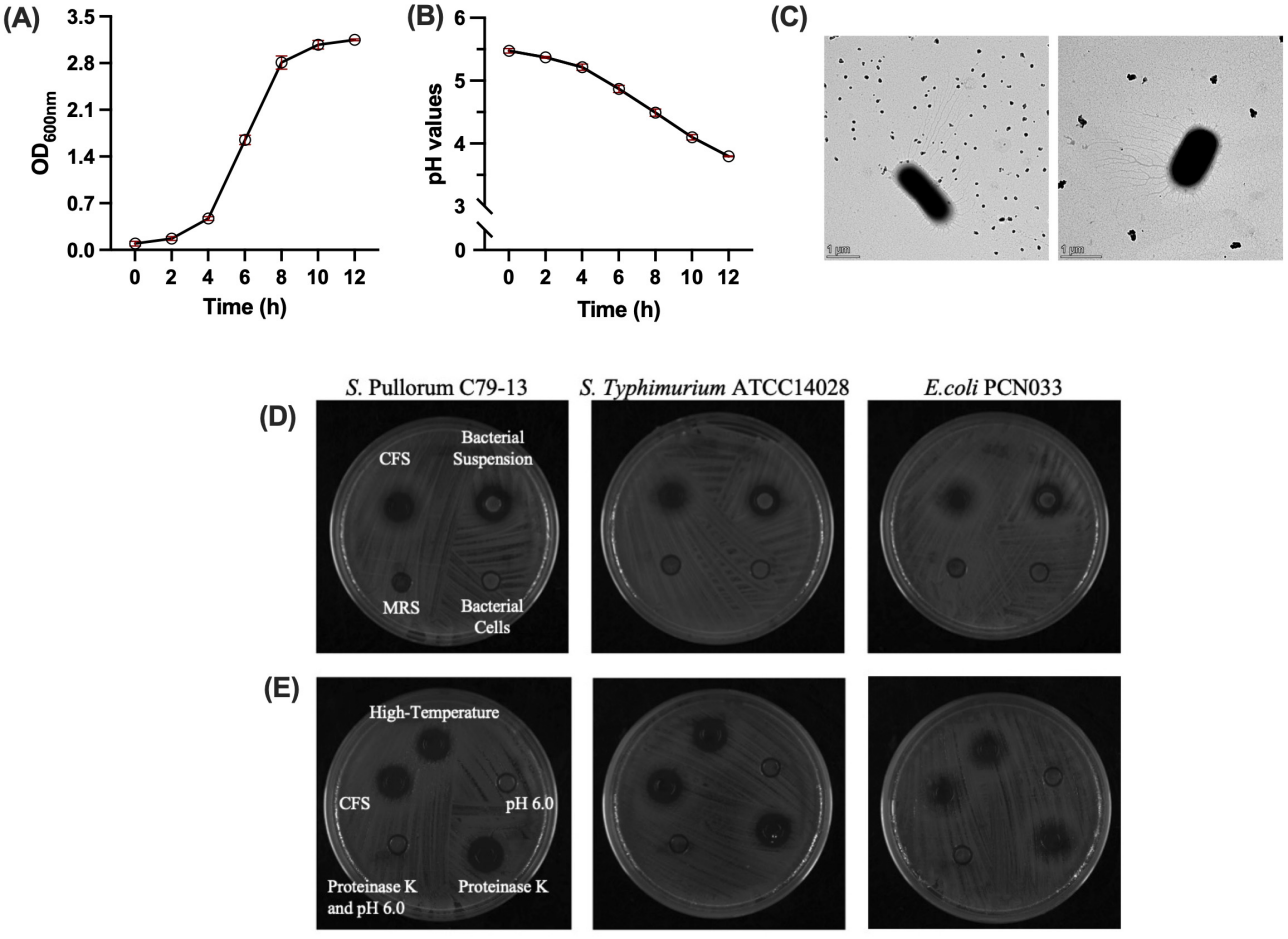
Characterization of growth properties, morphological features, and *in vitro* antimicrobial activity of *L. agilis* R22 Strain. **(A)** Growth capacity. **(B)** Acid production capacity. **(C)** Morphology of *L. agilis R22* under TEM. **(D)** Zones of inhibition from bacterial cell and cell-free supernatants. **(E)** Zones of inhibition from cell-free supernatants after different treatments.

*L. agilis* R22 entered the logarithmic growth phase at approximately 2 h and reached the stationary phase at around 8 h in MRS broth (Figure 1A). The pH of the culture medium continued to decrease with *L. agilis* R22 growth concurrently, reaching a minimum value of 3.80 at 12 h (Figure 1B). Observation by TEM revealed that *L. agilis* R22 exhibited a typical rod-shaped morphology. A single polar flagellum and peritrichous fimbriae were observed on the cell surface, indicating its potential for motility and adhesion (Figure 1C). In simulated gastrointestinal environments, the survival rate of *L. agilis* R22 reached 65%, and its antibacterial activity was comparable to that of the other strains tested (Supplementary Figure S1).

### 3.2 Antimicrobial activity and active substance characterization of *L. agilis* R22

The CFS of *L. agilis* R22 exhibited an antimicrobial activity comparable to that of the suspension, whereas the bacteria cells showed no inhibitory activity, suggesting that the active substance was primarily secreted into the supernatant (Figure 1D).

To further characterize the active substances, the CFS was subjected to different physicochemical treatments. We found that the antimicrobial activity of *L. agilis* R22 is strictly pH-dependent, as neutralizing the CFS to pH 6.0 resulted in a complete loss of inhibition. However, the activity remained unaffected by heat treatment or Proteinase K digestion. Together, these results indicate that the active components are heat-stable and non-proteinaceous, suggesting the inhibitory effect is primarily attributable to organic acids or similar acidic metabolites, rather than bacteriocins or heat-labile proteins.

After neutralization the pH to 6.0, the CFS completely lost its antimicrobial activity. In contrast, neither heat treatment nor digestion with Proteinase K could affect its inhibitory efficacy (Figure 1E). These results indicate that the antimicrobial activity of *L. agilis* R22 is primarily attributable to the substances produced in the supernatant.

### 3.3 Antibiotic susceptibility profile of *L. agilis* R22

*L. agilis* R22 was found to be susceptible to ampicillin, clindamycin, tetracycline, chloramphenicol, and erythromycin, while exhibited resistance to vancomycin, gentamicin, kanamycin, and streptomycin (Table 2). This typical intrinsic resistance pattern implies the absence of acquired transmissible resistance genes, indicating a favorable safety profile for probiotic application.

**Table 2:** Drug susceptibility of *L. agilis* R22.

Antibiotics	Boundary point (μg/mL)	MIC (μg/mL)	Drug susceptibility
Ampicillin	4	1	S
Vancomycin	n.r.	>32	R
Gentamicin	16	>32	R
Kanamycin	64	>128	R
Streptomycin	64	>128	R
Clindamycin	1	1	S
Tetracycline	8	1	S
Chloramphenicol	4	4	S
Erythromycin	1	1	S

R, Resistant; S, Susceptible; n.r., Not required.

### 3.4 Analysis of *L. agilis* R22 genome sequence

#### 3.4.1 General genome features and taxonomic identification

The genome sequencing and *de novo* assembly of *L. agilis* R22 yielded a draft genome consisting of 26 scaffolds, with a total size of 2,127,515 bp and a GC content of 41.62%. A total of 2,035 CDSs, 78 tRNA genes, and 3 rRNA genes were predicted. The overall architecture of the genome was visualized using a Circos plot (Supplementary Figure S2A).

The phylogenetic tree showed that strain R22 clustered within the same branch as *Ligilactobacillus agilis* (GCF_001436215.1), indicating its closest phylogenetic relationship to this species (Supplementary Figure S2B). Further confirmation was obtained by calculating the ANI between strain R22 and 14 reference strains (Supplementary Figure S2C). The ANI value against the *L. agilis* type strain was well-above the 95% species threshold, thus identifying the R22 as *Ligilactobacillus agilis*

#### 3.4.2 Functional annotation of the genome

To explore the genomic potential of *L. agilis* R22, its predicted CDSs were functionally annotated against multiple databases (Supplementary Table S1). KEGG pathway analysis revealed the primary metabolic features of *L. agilis* R22 (Supplementary Figure S3). At KEGG level 2, excluding “Global and overview maps,” the most abundant gene categories were concentrated in Carbohydrate Metabolism (163 genes), Amino Acid Metabolism (104 genes), and Membrane Transport (112 genes). Additionally, genes associated with the Metabolism of Cofactors and Vitamins (82 genes) and Glycan Biosynthesis and Metabolism (77 genes) were also highly enriched. These findings indicate that *L. agilis* R22 possesses a robust genetic capacity for nutrient utilization and environmental adaptation.

#### 3.4.3 Mining of probiotic-associated genes

Genes associated with probiotic functions in *L. agilis* R22 were systematically mined (Supplementary Table S2). A variety of genes related to environmental stress tolerance were found, including those responsive to acid, bile salt, heavy metal, temperature osmotic and oxidative stress. Additionally, a series of genes involved in host-microbe interactions were identified, such as those encoding adhesins and immunomodulatory factors. Notably, the genome of *L. agilis* R22 was found to contain a complete set of genes associated with motility and chemotaxis, including genes for flagellin, chemoreceptors, and chemotaxis proteins. This finding provides a molecular basis for the motile structures observed by TEM and suggests that *L. agilis* R22 may occupy a competitive niche within the gut through active motility.

#### 3.4.4 Analysis of virulence and antibiotic resistance genes

No known virulence factors nor pathogenicity-associated sequences were predicted in this genome following searching within the VFDB and PathogenFinder databases (homology >80%, coverage >80%). Similarly, no ARGs were detected through comparisons with the CARD and ResFinder databases. These findings provide initial evidence supporting the safety of *L. agilis* R22 as a probiotic candidate.

### 3.5 Analysis of the secretory metabolic profile of *L. agilis* R22

#### 3.5.1 Metabolite overview and PLS-DA

To investigate the metabolic activity of *L. agilis* R22 during different growth phases, an untargeted metabolomics analysis was performed using its CFS collected during the mid-logarithmic and stationary phases. In total, 1,550 and 1,514 metabolites were identified in the positive and negative ion modes, respectively (Supplementary Table S3).

**Figure 2:**
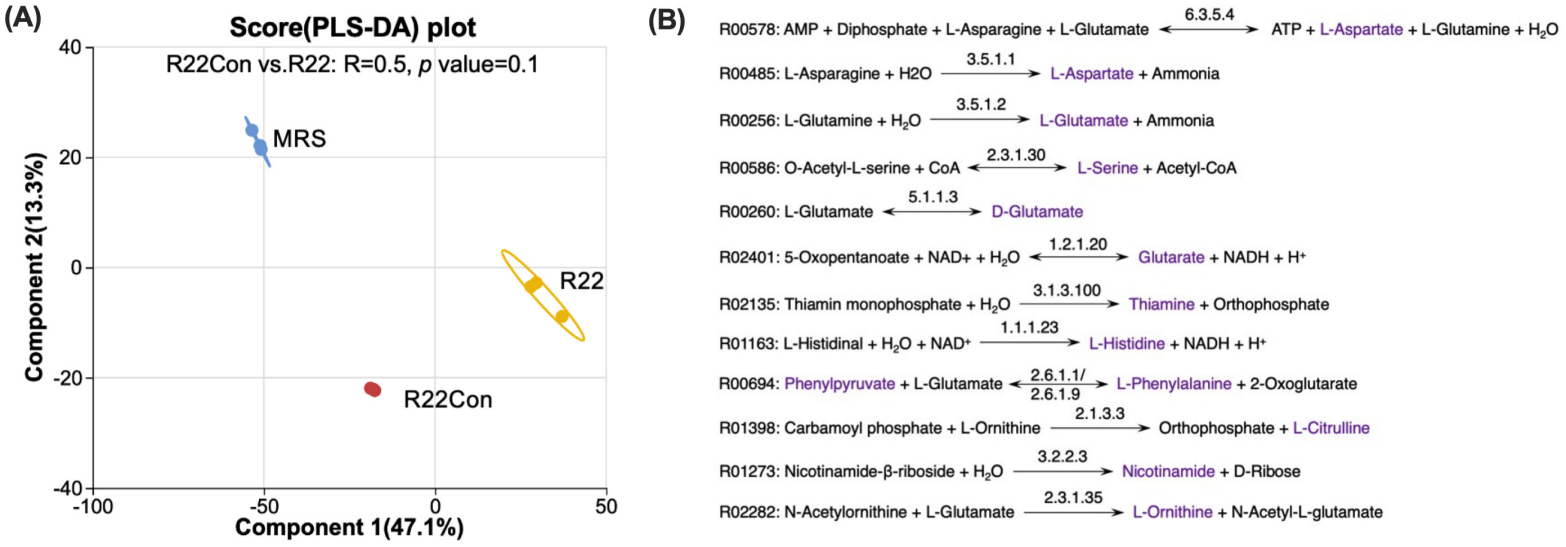
Analysis of R22 CFS by non-targeted metabolomics using PLS-DA and its direct pathways for nutrient synthesis. **(A)** PLS-DA analysis (mix) of CFS. **(B)** Direct pathways of nutrient synthesis. R22Con: CFS samples at the mid-logarithmic phase. R22: CFS samples at the stationary phase. MRS: the medium control.

The resulting PLS-DA plot revealed a separation trend between the mid-logarithmic and stationary phase samples, suggesting compositional differences (Figure 2A). However, this separation was not statistically significant according to the ANOSIM test (*R* = 0.5, *p* = 0.1). This result indicates that the core secretory metabolic profile of *L. agilis* R22 remained relatively stable between these two growth phases.

#### 3.5.2 Nutrient biosynthesis capacity

The capacity of *L. agilis* R22 to synthesize nutrients was investigated by integrating genomic and untargeted metabolomics data. By mapping the metabolites identified in the CFS against the biosynthetic enzyme-coding genes predicted in the genome within KEGG pathways, several key nutrient biosynthesis pathways were identified (Figure 2B). These included several essential amino acids for poultry (histidine and phenylalanine), non-essential amino acids (aspartate, glutamate, serine, citrulline and ornithine), thiamine (vitamin B1), and nicotinamide (a precursor to vitamin B3). For these pathways, the final amino acid products were consistently detected in the metabolome, while the genes encoding the enzymes for all key catalytic steps were identified in the genome.

Notably, for several other metabolic pathways, the final products were also detected in the CFS, and their corresponding key biosynthetic genes were present in the genome, even though their metabolic intermediates were not detected in the supernatant, likely due to their involvement in core intracellular metabolism. These nutrients included the essential amino acids (lysine, threonine, isoleucine, and tyrosine), the non-essential amino acids (alanine, proline and serine) and the succinate (Supplementary Table S4). This provides evidence at the metabolite level for the probiotic effects of *L. agilis* R22 within the host.

#### *3.6 L. agilis* R22 improves the growth performance of broilers

#### 3.6.1 Effects on growth performance and growth-related hormones

The effect of *L. agilis* R22 on the growth performance of broilers was assessed (Table 3). On day 14, the R22 group exhibited a significantly higher BW compared to the control group (*p* < 0.05). A numerical trend toward increased BW was also observed in the R22 group on days 21 and 40. Over the entire feeding period (days 1–40), the FCR in the R22 group was 0.05 lower than that of the control group. These results indicate that dietary supplementation with *L. agilis* R22 can effectively enhance the growth performance of broilers.

**Table 3:** Growth performance of broilers.

Items	Con	R22
BW, g		
d 1	46.56 ± 1.29	45.63 ± 1.16
d 14	451.56 ± 13.75^a^	461.63 ± 4.40^b^
d 21	773.35 ± 31.63	776.79 ± 35.82
d 40	2,482.19 ± 154.85	2,495.85 ± 102.95
ADG, g/d, per bird		
d 1–14	34.71 ± 1.20	34.84 ± 0.18
d 1–21	45.99 ± 1.37	45.76 ± 1.22
d 1–40	90.86 ± 7.31	88.95 ± 2.67
d 15–21	68.54 ± 4.04	67.59 ± 3.68
d 15–40	121.09 ± 11.04	118.08 ± 4.10
d 22–40	140.45 ± 15.37	136.69 ± 5.66
ADFI, g/d, per bird		
d 1–14	28.93 ± 0.98	29.71 ± 0.37
d 1–21	34.61 ± 1.53	34.82 ± 1.73
d 1–40	60.89 ± 3.87	61.26 ± 2.57
d 15–21	45.97 ± 5.62	45.02 ± 5.03
d 15–40	78.1 ± 5.86	78.24 ± 3.96
d 22–40	89.94 ± 8.50	90.48 ± 4.81
FCR, g/g		
d 1–14	1.20 ± 0.02	1.17 ± 0.02
d 1–21	1.33 ± 0.05	1.32 ± 0.04
d 1–40	1.5 ± 0.13	1.45 ± 0.05
d 15–21	1.51 ± 0.15	1.51 ± 0.10
d 15–40	1.56 ± 0.15	1.51 ± 0.07
d 22–40	1.57 ± 0.18	1.51 ± 0.08

Different superscripts in the same row showed significant difference. Con group: basal diets. R22 group: L. agilis R22 in diet. BW, Body Weight; ADG, average daily gain; ADFI, average daily feed intake; F/G, feed conversion ratio.

**Figure 3:**
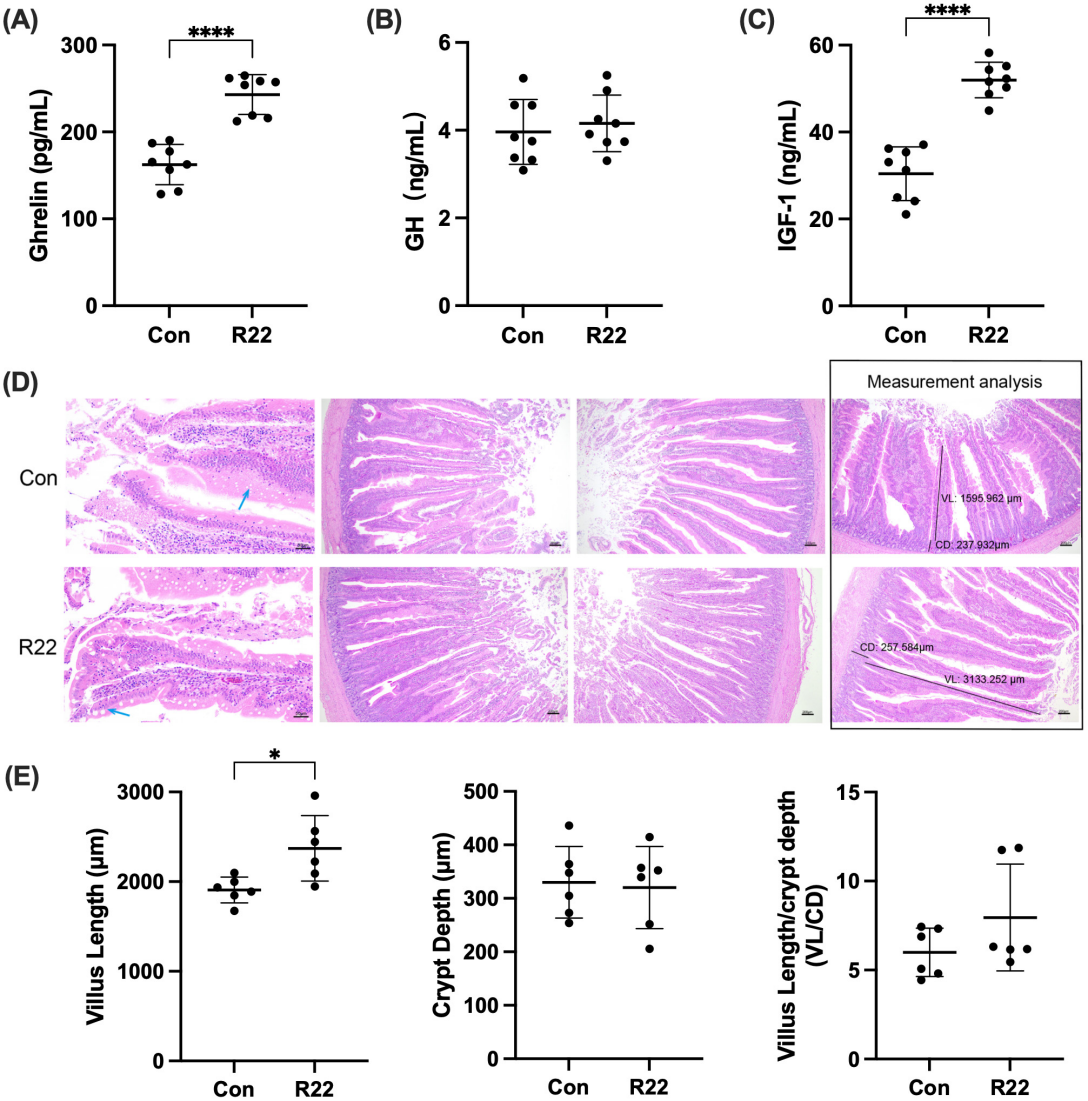
Effects of *L. agilis* R22 on growth-related hormone levels and intestinal morphology of broilers. **(A)** Ghrelin levels. **(B)** GH levels. **(C)** IGF-1 levels. **(D)** Intestinal morphology of duodenum. Blue arrow: goblet cell. **(E)** Duodenal Enterometry Analysis. Data are presented as mean ± SD. *p* < 0.05, ^*^p < 0.05, ^**^p < 0.00, ^***^p < 0.001 and ^****^p < 0.0001 vs. control group.

Then the serum levels of three key growth-related hormones were measured on day 40. Compared to the control group, the serum levels of both ghrelin and IGF-1 were significantly elevated in the R22 group (*p* < 0.0001; Figures 3A,C). No significant difference was observed in the levels of GH between the two groups (Figure 3B). These findings suggest that *L. agilis* R22 promote broiler growth by upregulating the secretion of ghrelin and IGF-1.

#### 3.6.2 Duodenal morphology

Histological examination of the duodenum in the R22 group revealed intact and well-structured villi with no apparent pathological lesions, and the goblet cells were well-preserved and exhibited the typical distended morphology (Figure 3D). Morphometric analysis further substantiated that the VL in the R22 group was significantly increased compared to the control group (*p* < 0.05; Figure 3E). These results indicate that dietary supplementation with *L. agilis* R22 enhances the intestinal absorptive surface area in the duodenum.

#### 3.6.3 Gut microbiota composition

**Figure 4:**
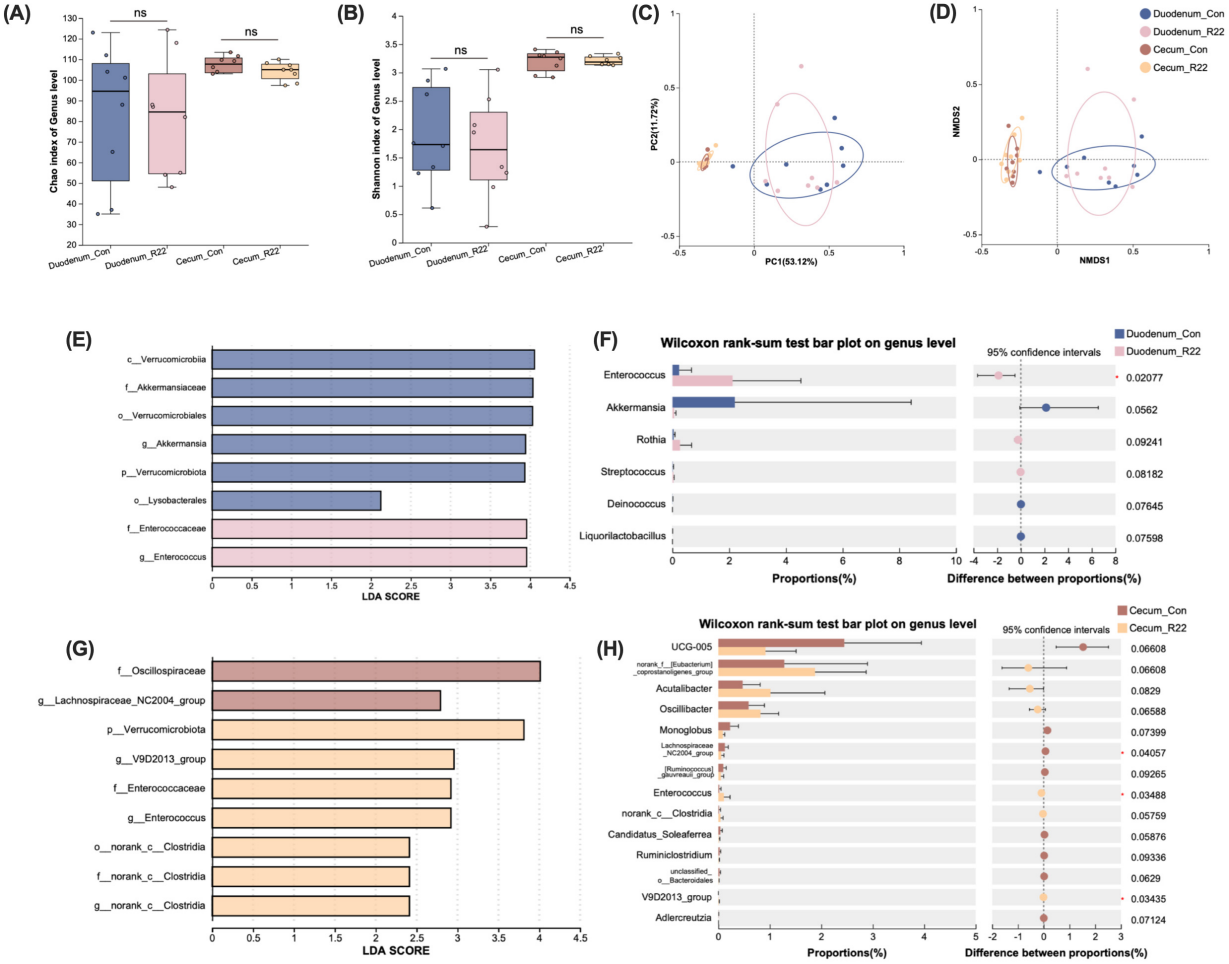
Gut microbiota composition of broilers fed with *L. agilis* R22. **(A)** Chao1 index. **(B)** Shannon index. **(C)** PCoA analysis. **(D)** NMDS analysis. **(E,F)** LEfSe analysis. **(G,H)** Changes at the genus level. Data are presented as mean ± SD. *p* < 0.05, ^*^p < 0.05, ^**^p < 0.00, ^***^p < 0.001 and ^****^p < 0.0001 vs. control group.

Alpha diversity analysis revealed no statistically significant differences in either the community richness (Chao1 index) or diversity (Shannon index) between the R22 and control groups in both the duodenum and the cecum (Figures 4A,B). Similarly, beta diversity analysis showed no distinct clustering between the two groups through PCoA and NMDS, indicating similar community structures (Figures 4C,D). These observations were further confirmed by ANOSIM and Adonis analyses, indicating that the feeding with *L. agilis* R22 did not significantly alter the overall structure of the gut microbiota in broilers (Supplementary Table S5).

The composition of top 10 microbes was analyzed at the phylum and genus levels (Supplementary Figure S4). At the phylum level, the gut microbiota in both the duodenum and cecum was predominantly composed of *Bacillota, Pseudomonadota*, and *Bacteroidota*. At the genus level, distinct differences were observed between the two intestinal segments. The duodenum was dominated by *Ligilactobacillus, Lactobacillus*, and *Sphingomonas*, whereas the cecal microbiota exhibited a more complex composition, with *Alistipes, Mediterraneibacter, Faecalibacterium*, and *Blautia* being the most abundant genera.

Although *L. agilis* R22 did not induce a major shift in the gut microbiota structure, further analysis revealed that its supplementation significantly impacted the abundance of specific taxa. LEfSe identified several bacterial taxa that were differentially enriched between the two groups (Figures 4E,F). To further investigate these differences, the relative abundances at the genus level were compared (Figures 4G,H). In the duodenum, the relative abundance of *Enterococcus* was significantly higher in the R22 group compared to the control group (*p* < 0.05). Additionally, the abundances of *Rothia* and *Streptococcus* showed a trend toward an increase in the R22 group (0.05 < *p* < 0.1). In the cecum, the R22 group exhibited significantly higher relative abundances of *Enterococcus* and V9D2013_group (*p* < 0.05), and a significantly lower abundance of *Lachnospiraceae*_NC2004_group (*p* < 0.05). Notably, although the strain R22 was identified as a member of the genus *Ligilactobacillus*, its administration did not lead to a significant change in the total abundance of this genus in either the duodenum or the cecum (*p* > 0.05; Supplementary Figure S5). This suggests that the exogenous *L. agilis* R22 may exert its effects primarily by modulating the existing microbial community rather than through its own extensive colonization.

#### 3.6.4 Intestinal metabolic profile

Untargeted metabolomics analysis of duodenal and cecal contents identified a total of 3,498 metabolites (Supplementary Table S6). PLS-DA revealed inherent differences between the metabolic profiles of the two intestinal segments, as well as a clear separation between the R22 and control groups within each segment (Supplementary Figure S6). This separation results showed a significant difference in the metabolic profiles between the two groups in the duodenum (*R* = 0.2606, *p* = 0.01).

**Figure 5:**
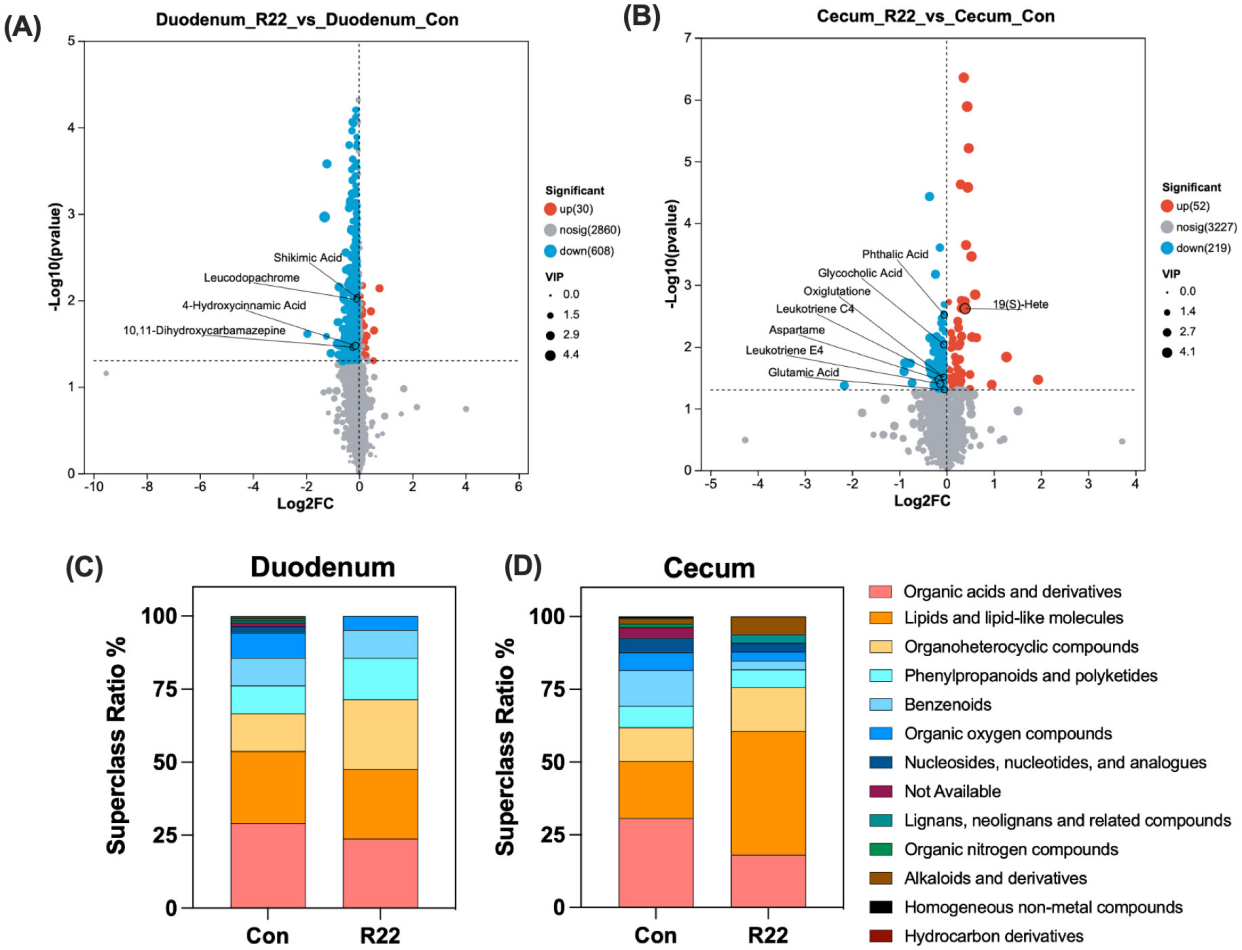
Significant differential metabolite analysis (mix). **(A,B)** volcano plot of significantly different metabolites in the duodenum and cecum. **(C,D)** HMDB compound percentage statistics at superclass level

Based on the OPLS-DA model, key differential metabolites between the two groups were identified. In the duodenum, a total of 638 differential metabolites were identified, of which 30 were upregulated and 608 were downregulated in the R22 group (Figure 5A). Chemical classification of these metabolites at the Superclass level using the HMDB database revealed that the upregulated metabolites in the R22 group were predominantly classified as organoheterocyclic compounds (27.8%), phenylpropanoids and polyketides (14.3%), and organic oxygen compounds (4.76%; Figure 5C). In the cecum, 271 differential metabolites were identified, with 52 being upregulated and 219 downregulated in the R22 group (Figure 5B). In contrast to the duodenum, the upregulated metabolites in the cecum were primarily classified as lipids and lipid-like molecules (42.4%), organic acids and derivatives (18.2%), and benzenoids (3.03%; Figure 5D). These findings suggest that *L. agilis* R22 may exert its functions by modulating distinct classes of metabolites in different intestinal segments.

To understand the metabolic pathways in which these differential metabolites are involved, the KEGG pathway enrichment analysis was performed. In the duodenum, a total of seven pathways were significantly enriched (*p* < 0.05; Supplementary Figure S7A). Within these pathways, four key metabolites-−10,11-dihydroxycarbamazepine, leucodopachrome, 4-hydroxycinnamic acid, and shikimic acid—were significantly decreased in the R22 group (*p* < 0.05; Supplementary Figure S7B). In the cecum, sixteen pathways were found to be significantly enriched (*p* < 0.05; Supplementary Figure S8A). Notably, several pathways closely related to immune and inflammatory regulation were significantly impacted, such as arachidonic acid metabolism, glutathione metabolism, and the bile secretion pathway. With the exception of 19(S)-HETE, seven other key differential metabolites within these pathways were significantly downregulated (*p* < 0.05; Supplementary Figure S8B).

### 3.7 *L. agilis* R22 provides protection against *Salmonella* infection in chicks

There were no significant differences in BW among the groups prior to the challenge of *S*. Pullorum. Following infection, the growth of the chicks in the SP group was significantly inhibited, with their body weight being significantly lower than that of the unchallenged NC group (*p* < 0.05). Crucially, supplementation with *L. agilis* R22 significantly mitigated this effect, resulting in significantly higher BW in the R22 group compared with the SP group from days 5 to 9 (*p* < 0.05, Table 4). No mortality was observed in any group throughout the experimental period. These results indicate that *L. agilis* R22 effectively alleviates growth retardation in chicks caused by *S*. Pullorum infection.

**Table 4:** Changes in the BW (g) of chicks before and after *S*. Pullorum infection.

Group/days	NC	SP	R22
1d	37.79 ± 1.92	39.53 ± 1.60	37.35 ± 1.63
2d	38.56 ± 1.38	38.45 ± 1.54	38.71 ± 1.64
3d	44.36 ± 1.73	44.35 ± 2.46	43.75 ± 2.76
4d (infected)	52.35 ± 2.06	51.47 ± 3.01	51.66 ± 2.81
5d	58.46 ± 2.52^a^	53.00 ± 3.86^b^	56.52 ± 3.01^c^
6d	64.12 ± 3.07^a^	55.79 ± 5.48^b^	62.41 ± 4.07^a^
7d	68.39 ± 3.37^a^	62.04 ± 6.72^b^	70.15 ± 5.21^a^
8d	79.75 ± 4.55^a^	73.10 ± 3.38^b^	80.13 ± 4.51^a^
9d	85.94 ± 4.21^a^	79.03 ± 4.43^b^	86.68 ± 4.22^a^

^a, b, c^ indicate that groups with different superscript letters within the same row have significant differences.

**Figure 6:**
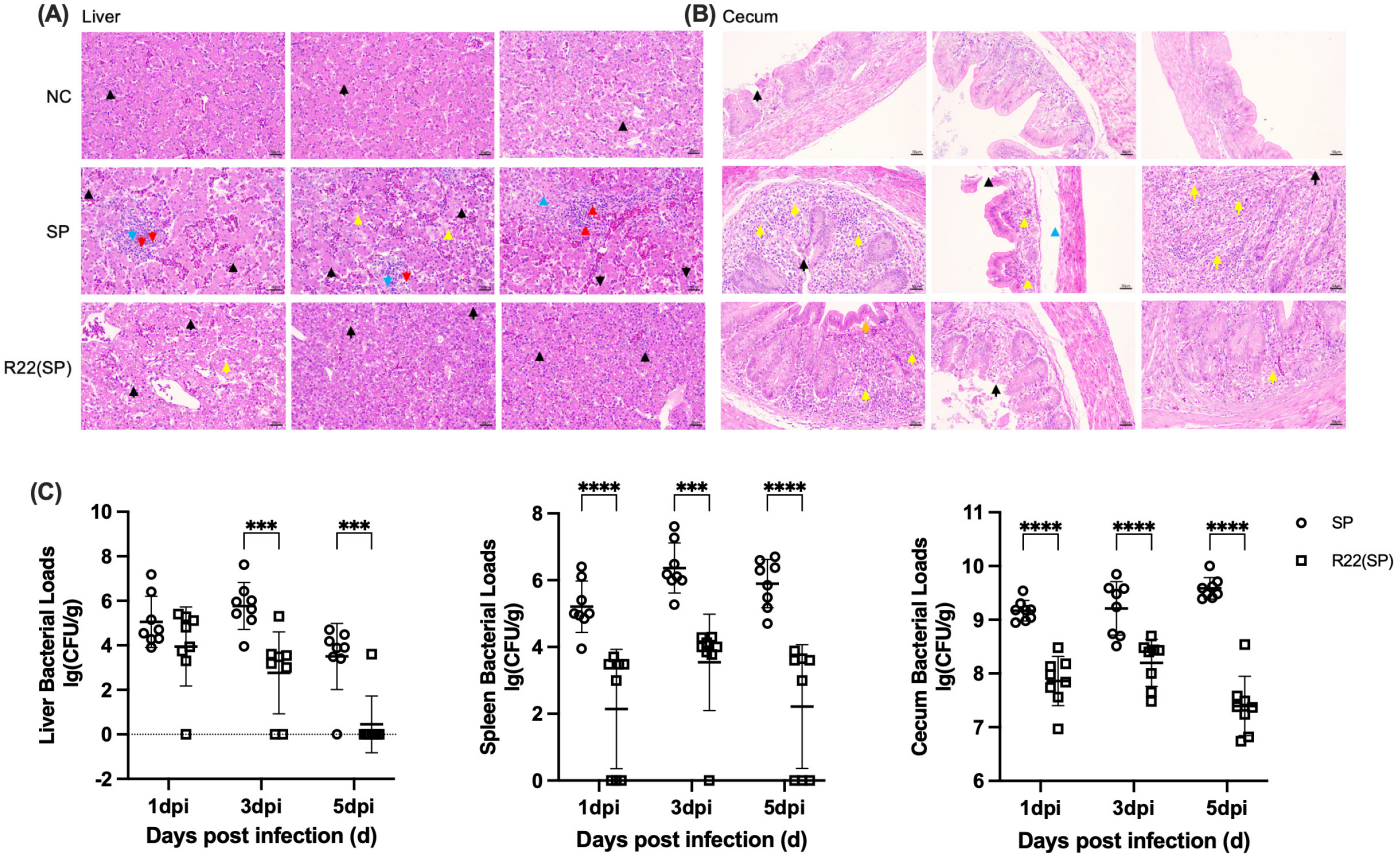
*L. agilis* R22 enhanced the resistance of chickens to *S*. Pullorum infection. **(A)** Pathological change in liver. Black arrow: lipid vacuoles. Yellow arrow: hepatocyte steatosis. Red arrow: steatopygic infiltration. Blue arrow: fibrosis. **(B)** Pathological changes in cecum. Black arrow: epithelial cell desquamation. Yellow arrow: loosely arranged crypts with reduced number. Blue arrow: submucosal edema. **(C)** Bacterial loads in liver, spleen and cecum. Data are presented as mean ± SD. *p < 0.05, **p < 0.01, ***p < 0.001, ****p < 0.0001 vs. control group.

Histopathological analysis of the liver and cecum was performed on day 5 post-infection, the tissues from the NC group exhibited normal histological structures (Figures 6A, B). In contrast, chicks in the SP group displayed severe pathological lesions. The liver was characterized by extensive inflammatory cell infiltration, hepatocyte swelling and steatosis. In the cecum, severe damage was observed, including shedding of the mucosal epithelium, destruction of the crypt architecture and submucosal edema. Remarkably, these pathological damages were significantly ameliorated in the R22 group. The tissue structure of the liver and cecum in this group remained largely intact, with only mild inflammatory cell infiltration observed. These findings indicate that *L. agilis* R22 effectively preserves the tissue integrity of chicks caused by *S*. Pullorum infection.

To dynamically monitor the pathogen loads, quantitative analysis was performed on *S*. Pullorum in tissues from infected chicks. On day 2 and day 5 post-infection, the bacterial loads in the cecum, liver, and spleen of the R22 group were significantly lower than those in the SP group *(p* < 0.001; Figure 6C). These results demonstrate that *L. agilis* R22 not only inhibits the colonization of *S*. Pullorum in the intestine but also restricts its systemic dissemination to the liver and spleen, thereby contributing to the observed alleviation of histopathological damage.

### 3.8 The protective effect of *L. agilis* R22 can be mediated by the gut microbiota

**Figure 7:**
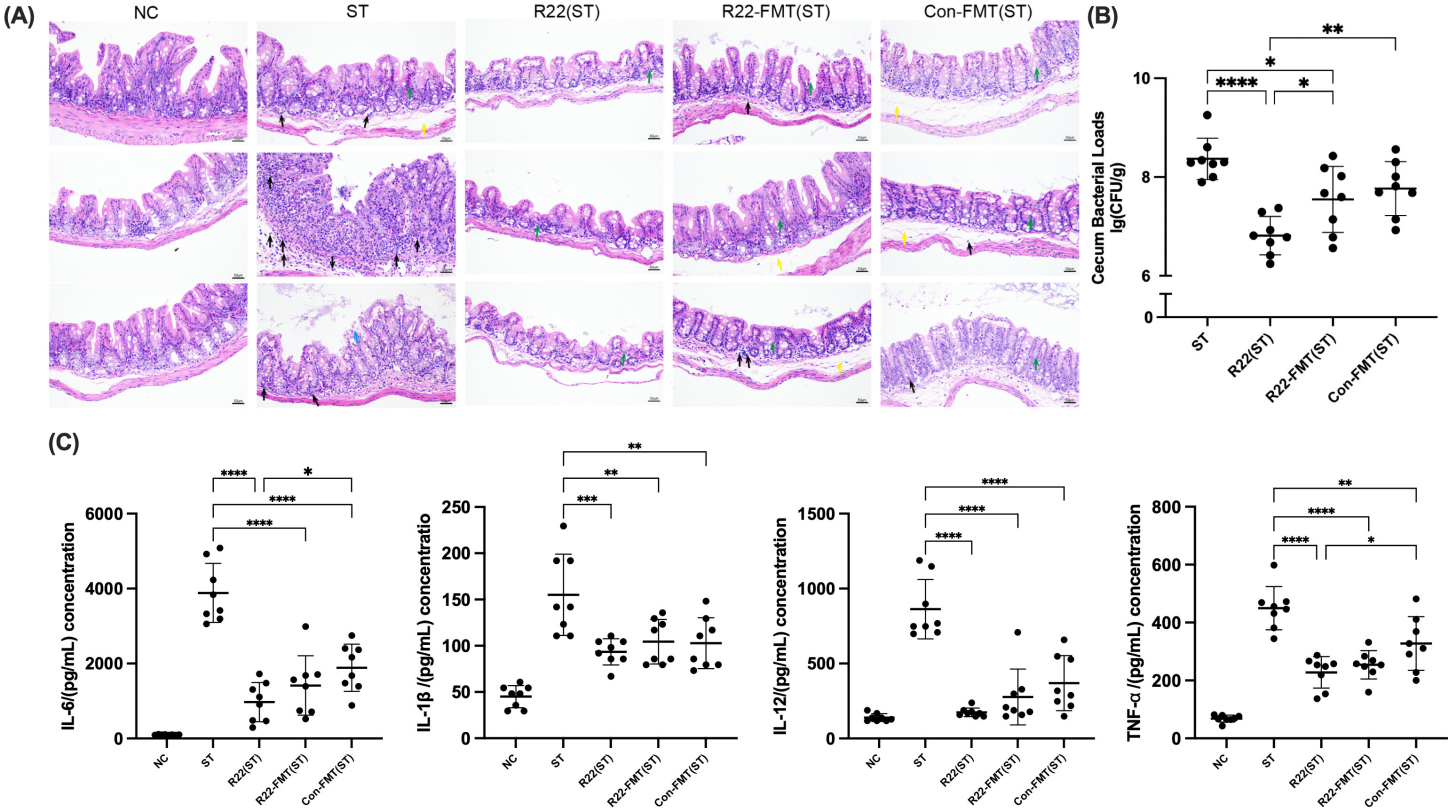
Resistance effect of FMT in mice infected with *S*. Typhimurium. **(A)** Pathological change in cecum. **(B)** Bacterial loads in the cecum. **(C)** Levels of inflammatory cytokines in the serum. Black arrow: inflammatory cell infiltration. Yellow arrow: submucosal edema. Green arrow: thinning of mucosal structures. Blue arrow: desquamation of mucosal epithelial cells. Data are presented as mean ± SD. *p* < 0.05, ^*^p < 0.05, ^**^p < 0.00, ^***^p < 0.001 and ^****^p < 0.0001 vs. control group.

To determine whether the probiotic function of *L. agilis* R22 can be mediated by the gut microbiota it remodels, a FMT experiment was conducted. Fecal slurries from donor mice that were either treated with *L. agilis* R22 or untreated were transplanted into newly weaned recipient mice, which were subsequently challenged with *S*. Typhimurium. Mice in the ST group exhibited a significantly lower BW compared to the NC group on 3 days post-infection (*p* < 0.05; Table 5). In contrast, the BW of mice in the R22-FMT (ST) group was not only higher than that of the ST group but also comparable to that of the R22 (ST) group, which received R22 directly (Table 5). Histopathological analysis revealed that the cecal mucosal in the ST group was severely disrupted, accompanied by massive inflammatory cell infiltration. In contrast, the tissue damage in both the R22 (ST) and R22-FMT (ST) groups was significantly attenuated, with the cecal structure remaining largely intact (Figure 7A).

**Table 5:** Changes in the BWR of mice after *S*. Typhimurium infection.

Group/days post infection	NC	ST	R22(ST)	R22-FMT(ST)	Con-FMT(ST)
1d	1.77 ± 0.10	1.65 ± 0.11	1.69 ± 0.03	1.80 ± 0.14	1.72 ± 0.11
2d	1.85 ± 0.12	1.71 ± 0.07	1.77 ± 0.03	1.82 ± 0.14	1.80 ± 0.11
3d	1.92 ± 0.12^a^	1.72 ± 0.08^b^	1.80 ± 0.04^ab^	1.82 ± 0.13^ab^	1.85 ± 0.12^ab^
4d	1.97 ± 0.13^a^	1.67 ± 0.07^b^	1.77 ± 0.04^b^	1.80 ± 0.16^b^	1.80 ± 0.17^b^
5d	2.00 ± 0.11^a^	1.61 ± 0.13^b^	1.76 ± 0.05^b^	1.75 ± 0.15^b^	1.77 ± 0.18^b^
6d	2.08 ± 0.12^a^	1.57 ± 0.13^b^	1.72 ± 0.09^bc^	1.72 ± 0.18^bc^	1.73 ± 0.17^c^

BWR, Body weight ratio. ^a, b, c^ indicate that groups with different superscript letters within the same row have significant differences.

The bacterial loads of Salmonella in the cecum indicated that both the R22 (ST) group and the R22-FMT (ST) group showed a significant reduction compared to the ST group (*p* < 0.05; Figure 7B). Notably, although the pathogen loads in the mice receiving fecal microbiota from the Con-FMT (ST) group showed a downward trend, this reduction did not show significance compared to the ST group. This finding indicates that the microbiota specifically modulated by *L. agilis* R22 plays a critical role in inhibiting Salmonella colonization. Furthermore, a more pronounced inhibitory effect was observed in the R22 (ST) group compared to the R22-FMT (ST) group. This suggests that the protective effect may also be partially dependent on the direct presence of the strain and its metabolites.

Detection of pro-inflammatory cytokine levels in mouse serum revealed that *S*. Typhimurium infection caused significant increases in IL-6, IL-12, TNF-α, and IL-1β levels in the ST group (*p* < 0.0001; Figure 7C). Compared to the ST group, the levels of these pro-inflammatory cytokines were significantly downregulated in both the R22 (ST) and R22-FMT (ST) groups (*p* < 0.001). Notably, the R22-FMT (ST) group exhibited stronger inhibitory effects than the Con-FMT (ST) group, further indicating that the R22-modified microbiota possesses enhanced anti-*S*. Typhimurium and immunomodulatory capabilities.

## 4 Discussion

The development of safe and efficacious probiotics as antibiotic alternatives is important for safeguarding both poultry and public health (Shah et al., 2024). This study aims to isolate LAB strains with excellent probiotic potential from healthy chicken sources and conduct a systematic evaluation covering *in vitro* characteristics, multi-omics basis, *in vivo* efficacy, and core mechanisms of action. We successfully identified *L. agilis* R22 and confirmed its dual functions in promoting growth and combating infections.

### 4.1 Comprehensive evaluation of *L. agilis* R22 as a probiotic candidate

The screening process is the first critical step for the successful application of probiotics (Dowarah et al., 2017). An ideal strain must be able to tolerate the conditions of gastrointestinal tract and possess broad-spectrum antimicrobial activity (Markowiak and Sliżewska, 2018). In this study, *L. agilis* R22 demonstrated exceptional tolerance to acid and bile salts, providing physiological evidence for its ability to survive during intestinal transit and to successfully colonize. Regarding its antimicrobial activity, *L. agilis* R22 demonstrated potent inhibitory effects against several common poultry pathogens. Characterization experiments indicated that this antimicrobial activity is primarily derived from the production of organic acids. This finding is consistent with the core antimicrobial mechanism of LAB that involves lowering the intestinal pH and interfering with the cellular metabolism of pathogens.

Biosafety is a prerequisite for the commercialization of probiotics (Merenstein et al., 2023). The present study found that *L. agilis* R22 exhibited resistance to vancomycin and aminoglycosides (e.g., gentamicin and kanamycin). However, this resistance profile does not pose a safety concern, as it is well-documented as being intrinsic to the *Lactobacillus*. Extensive research has shown that *Lactobacillus* generally lack the cytochrome-dependent electron transport chain required for the energy-dependent uptake of aminoglycosides, leading to intrinsic resistance (Dec et al., 2020). Similarly, their cell wall peptidoglycan precursors often terminate in D-alanine-D-lactate (D-Ala-D-Lac) rather than the D-alanine-D-alanine (D-Ala-D-Ala), the target of vancomycin, resulting in natural insensitivity (Stogios and Savchenko, 2020). This type of resistance is therefore classified as intrinsic and non-transferable, a characteristic that aligns with the safety assessment guidelines for probiotics established by EFSA. Tetracycline and chloramphenicol resistance phenotypes and resistance genes are also widely present in many LAB. Although the phenotypes do not fully correspond to the genotypes, this serves as a reminder that the mechanisms of resistance in lactobacilli warrant further investigation (Dec et al., 2017; Anisimova and Yarullina, 2018). This conclusion is further strengthened by the whole-genome analysis, which confirmed that the genome of *L. agilis* R22 contains no known virulence factor genes nor antibiotic resistance genes located on mobile genetic elements. Collectively, these findings support the high biosafety profile of *L. agilis* R22 for its application as a feed additive.

### 4.2 The genetic and metabolic basis of *L. agilis* R22

Genomic analysis provided the genetic basis for several key probiotic traits of *L. agilis* R22, including its stress tolerance and adhesion potential. The analysis also revealed its motility, a finding that corroborates the flagellar structures observed by TEM. Among motile *Lactobacillus, L. agilis* and *L. ruminis* are the only species known to originate from the animal and human gut (Yu et al., 2017; Suzuki et al., 2020). This capacity for active motility may confer a unique ecological advantage upon *L. agilis* R22, such as enabling it to penetrate the mucus layer (Kajikawa et al., 2018), actively evade unfavorable conditions (Suzuki et al., 2023), and more effectively seek out adhesion sites (Stephenson et al., 2010), thereby facilitating more stable colonization. These genes ensure that the *L. agilis* R22 strain survives, colonizes, and maintains its adaptability in the inflamed gastrointestinal tract, thereby enabling the strain to effectively displace Salmonella and protect the intestinal mucosa.

Untargeted metabolomic analysis revealed that *L. agilis* R22 is capable of autonomously synthesizing a variety of essential and non-essential amino acids for poultry, as well as B vitamins, a trait shared with some other *Lactobacillus* (Feito et al., 2022; Karaseva et al., 2023; Sevillano et al., 2024). Succinate promotes mitochondrial energy metabolism in intestinal stem cells, enhancing cellular activity and thereby improving egg production performance in laying hens (Zhou et al., 2021). This finding suggests that *L. agilis* R22 not only improves the gut environment but may also directly provide the host with essential nutrients, thereby contributing to its growth-promoting effects.

It should be noted that while the integrated multi-omics analysis elucidates the nutrient synthesis capacity of *L. agilis R22*, metabolic profiles within the LAB are widely recognized to be highly strain-specific (Fuhren et al., 2020; Koduru et al., 2022). It remains to be fully defined which of these biosynthetic pathways are conserved features of the species and which are unique evolutionary adaptations of the *L. agilis* R22 isolate. Therefore, future comparative genomic and metabolomic analyses involving multiple reference strains are warranted to delineate the strain-specific metabolic signatures of *L. agilis* R22.

### 4.3 Dual *in vivo* function of *L. agilis* R22 in poultry

This study validated the dual function of *L. agilis* R22 in promoting growth and conferring protection against infection in chickens. Dietary supplementation with *L. agilis* R22 significantly enhanced the duodenal villus height, which effectively expands the absorptive surface area for nutrients. This finding is consistent with previous reports that LAB can promote growth by improving intestinal morphology (Yang et al., 2023; Zhou et al., 2025). Importantly, this enhanced nutrient absorption directly translated into positive trends in production efficiency. Although statistical significance was only observed for body weight at day 14, the *L. agilis* R22 group exhibited a notable 0.05 numerical reduction in the overall FCR (days 1–42). While such FCR improvements often fall short of statistical significance in small-scale pen trials due to limited replicate numbers, a 0.05 reduction is highly biologically and economically relevant in commercial broiler production. It represents substantial savings in feed costs and improved overall production efficiency, thereby underscoring the immense application potential of *L. agilis* R22 in the poultry industry.

The study also found that *L. agilis* R22 significantly upregulated the serum levels of ghrelin and IGF-1. Although ghrelin is traditionally known as an appetite-stimulating hormone (Geelissen et al., 2003; Tanaka et al., 2003), the feed intake of the broilers did not increase concomitantly in the present study. This apparent discrepancy can be explained by two main factors. First, the *L. agilis* R22 may have improved intestinal health and enhanced feed digestibility, allowing the broilers to meet their energetic demands for growth without requiring extra feed consumption. The satiety signals generated by efficient nutrient absorption may have counteracted the orexigenic effect of ghrelin (Lewiński et al., 2021). Second, in poultry, the physiological role of ghrelin extends beyond appetite regulation; it plays a crucial role in energy partitioning and growth modulation. The elevated ghrelin levels may not have primarily acted on the feeding centers, but rather served as a metabolic signal that, in concert with elevated IGF-1, redirected the absorbed nutrients toward muscle and skeletal growth (Vélez and Unniappan, 2020). Interestingly, the level of GH itself remained unchanged. A possible explanation is that the elevated IGF-1 suppressed pituitary GH secretion through a classical negative feedback loop, thereby maintaining GH homeostasis (Jia et al., 2018). These findings suggest that *L. agilis* R22 promotes broiler growth not via a single pathway of increasing feed intake, but through an endocrine regulatory network that systematically optimizes nutrient partitioning while maintaining stable feed consumption.

The capacity of *L. agilis* R22 to combat Salmonella infection mitigated the severe inflammatory damage to the cecum and liver by inhibiting pathogen colonization, thereby preserving tissue integrity. This was substantiated by the significantly lower Salmonella loads in the cecum, liver, and spleen of the R22-treated group at multiple time points post-infection. The reduction in the cecal pathogen loads signifies the establishment of a first line of defense (Neveling et al., 2020), where *L. agilis* R22 likely inhibits the initial colonization and proliferation of Salmonella through mechanisms such as acid production and competitive exclusion (He et al., 2024). More importantly, the concurrent reduction of bacterial loads in the liver and spleen provides strong evidence that *L. agilis* R22 effectively blocks the translocation of the pathogen from the gut into the bloodstream and its subsequent systemic dissemination.

Although the metabolizable energy level of the basal diet for days 1–21 (2,850 kcal/kg) was slightly lower than the 3,000 kcal/kg recommended by the NY/T 33-2004 standard, it reflects typical commercial production practices in the experimental region. Future studies may increase the metabolizable energy level above 3,000 kcal/kg to fully realize the maximum growth potential of broilers.

### 4.4 Microbiota modulation of *L. agilis* R22

Modulation of the gut microbiota is a primary mechanism through which LAB exert their beneficial effects (Muñoz-Tamayo et al., 2011; Varmuzova et al., 2016). FMT is an emerging technique capable of remodeling the gut microbiota, which has been shown to effectively improve host health (Sorbara and Pamer, 2022). In animal models, numerous studies have demonstrated that FMT can help establish the early-life microbiota in various young animals (Kim et al., 2021; Yan et al., 2021). Particularly in chickens, transplanting fecal matter from donors with superior growth performance can improve the intestinal barrier and promote growth in recipients by maintaining the Th17/Treg cell balance and reducing inflammation in the jejunum (Ma et al., 2023). Another study found that FMT from donors with low abdominal fat deposition could reduce excessive fat accumulation in recipients by regulating lipid metabolism (Chen et al., 2023). The present study found that mice in the R22-FMT (ST) group exhibited significantly attenuated intestinal pathological damages and a lower pathogen load after Salmonella infection compared to the infection-only group. Of particular importance, the protective effect in the R22-FMT (ST) group was significantly more potent than that observed in the group that received microbiota from untreated control donors. This comparison demonstrates that the microbiota specifically remodeled by *L. agilis* R22 plays a superior role in preventing infections, compared to a non-R22 treated microbial community.

## 5 Conclusion

In conclusion, this study identifies chicken-origin Ligilactobacillus agilis R22 as a safe and effective probiotic candidate. *In vitro*, R22 exhibited strong stress resistance, antibacterial activity, and acid production capacity. Genome sequencing revealed multiple probiotic-associated genes without virulence factors, along with pathways for synthesizing essential and non-essential amino acids, B vitamins, and organic acids beneficial for livestock and poultry. *In vivo*, R22 promoted broiler growth, conferred protection against Salmonella infection, and modulated gut microbiota composition and metabolites. Fecal microbiota transplantation (FMT) in a mouse model indicated that its protective effects are mediated by the gut microbiota. This work offers a promising strain for microbial feed additive development.

## Data Availability

The final genome assembly has been deposited in the NCBI GenBank database (accession number: PRJNA1194302). The raw sequencing data of 16S rRNA have been deposited in the NCBI Sequence Read Archive database (accession number PRJNA1263642).
