# Supplementary figures and images for: A chicken-origin Ligilactobacillus agilis R22 exerts probiotic features including growth-promotion and anti-Salmonella infection
Source: Front Microbiol. 2026 Jun 22;17:1862425. doi: 10.3389/fmicb.2026.1862425 (PMC13334848; doi:10.3389/fmicb.2026.1862425)

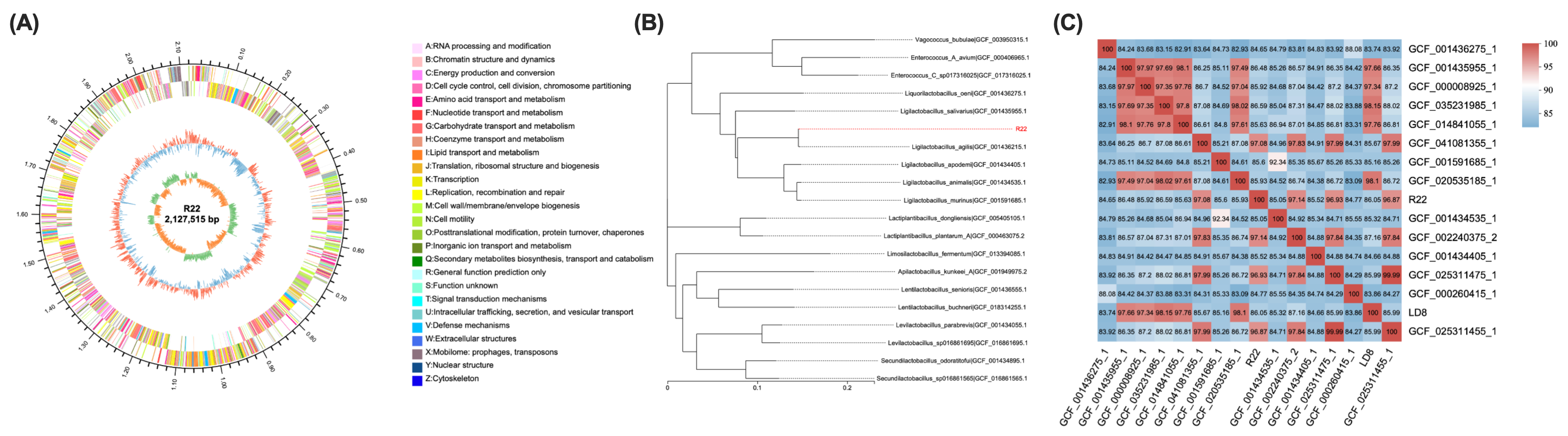

Supplement: Supplementary file 1 [file Image_1.tiff]

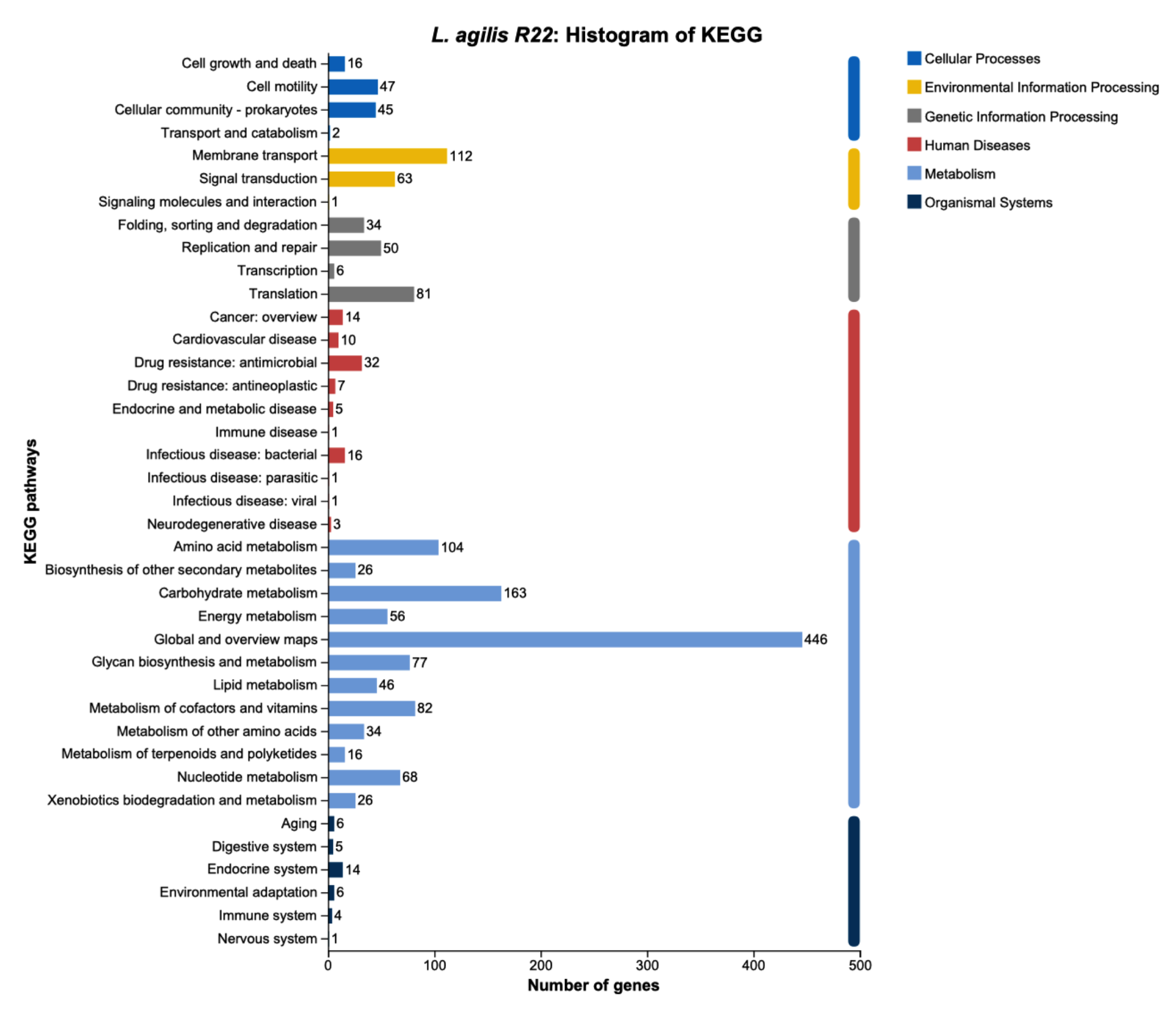

Supplement: Supplementary file 2 [file Image_2.tiff]

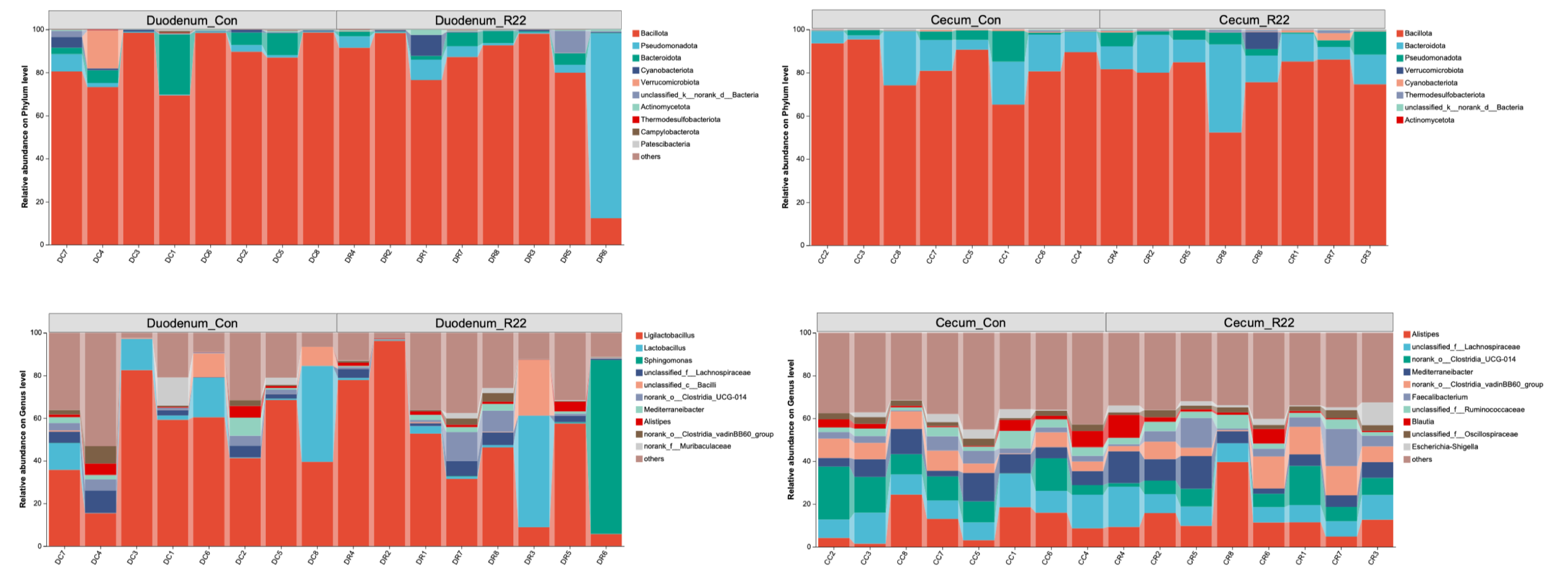

Supplement: Supplementary file 3 [file Image_3.tiff]

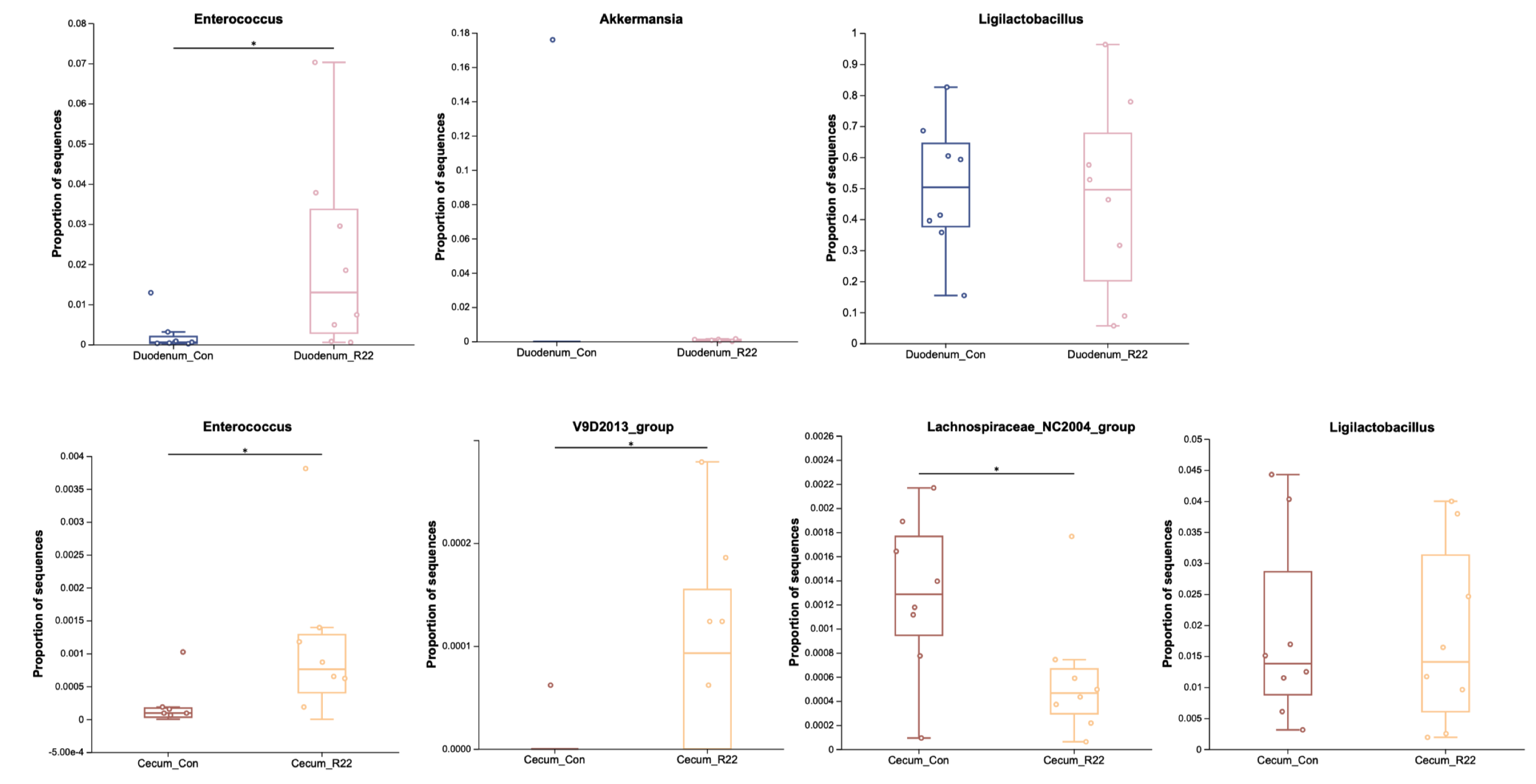

Supplement: Supplementary file 4 [file Image_4.tiff]

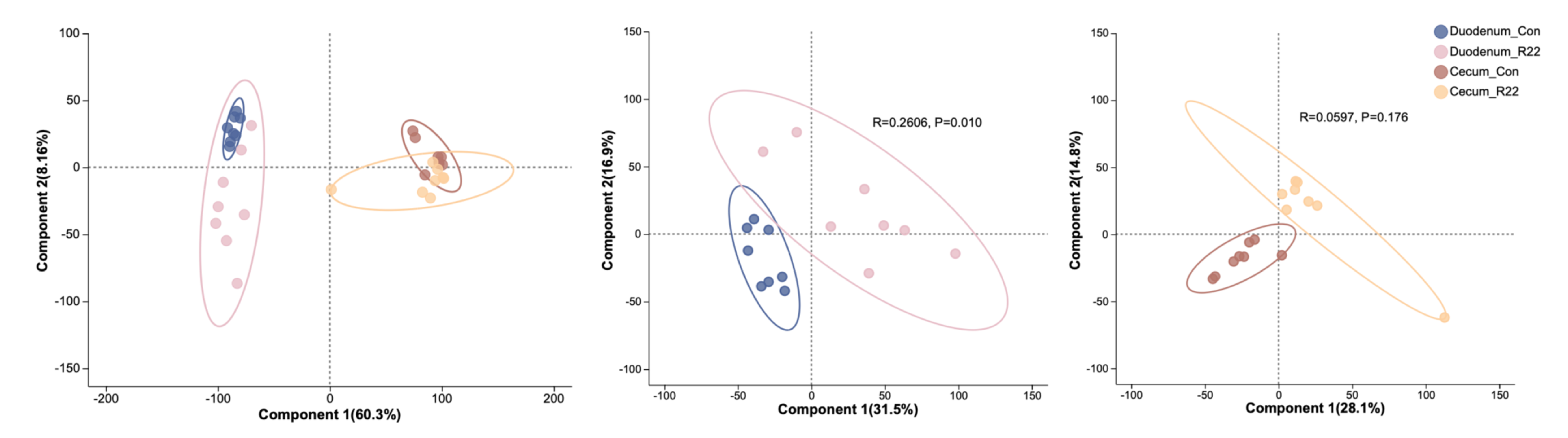

Supplement: Supplementary file 5 [file Image_5.tiff]

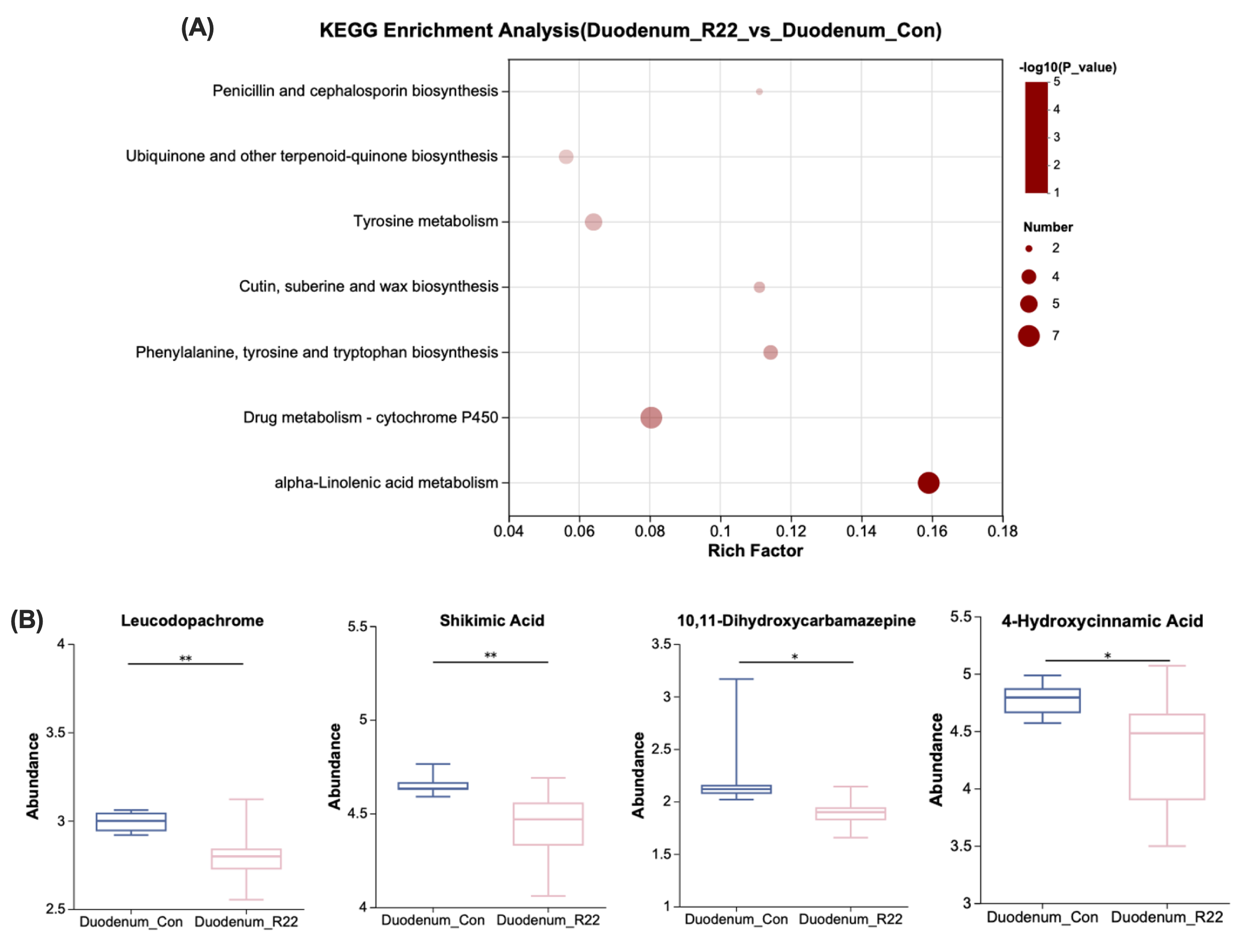

Supplement: Supplementary file 6 [file Image_6.tiff]

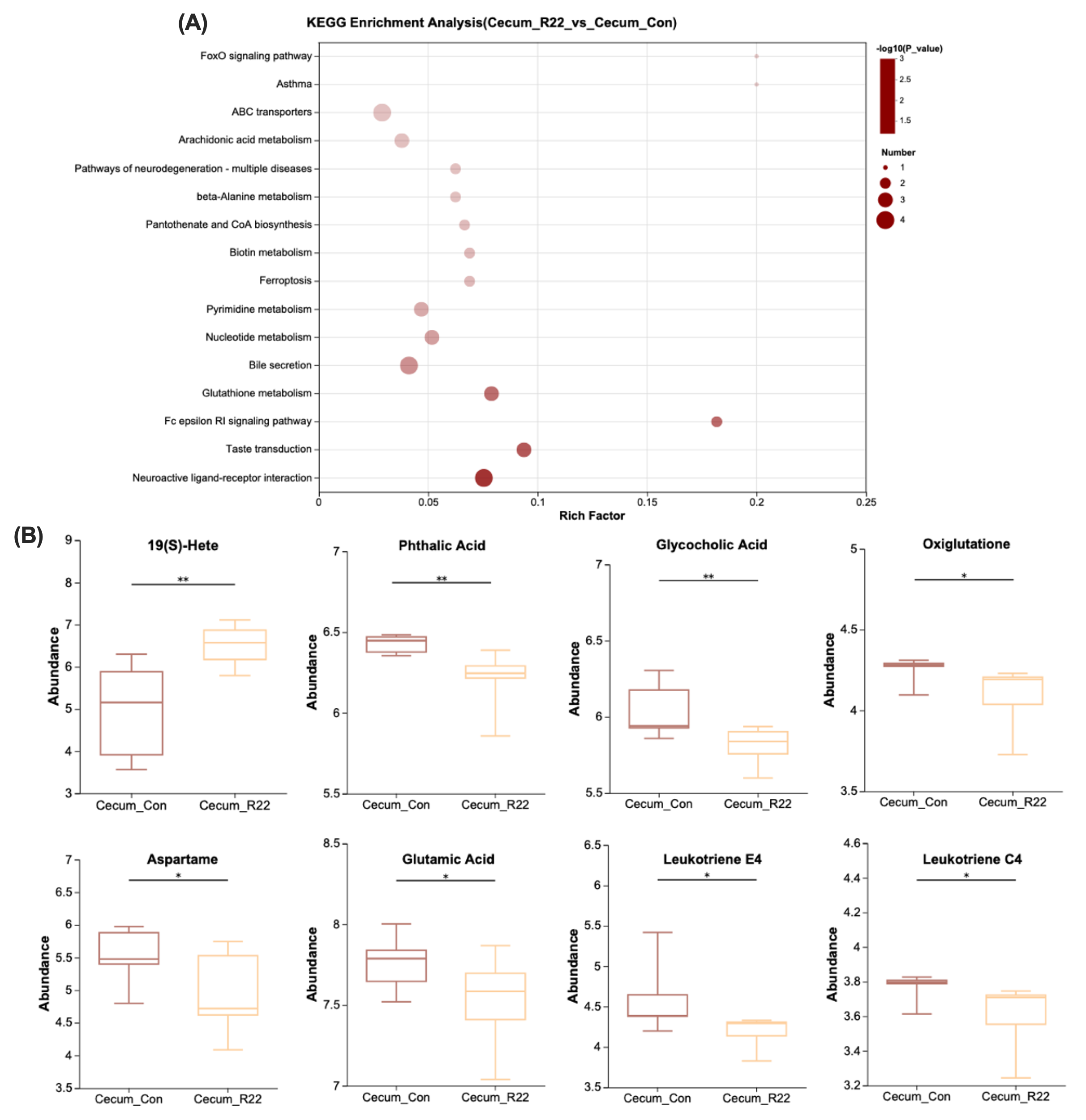

Supplement: Supplementary file 7 [file Image_7.tiff]

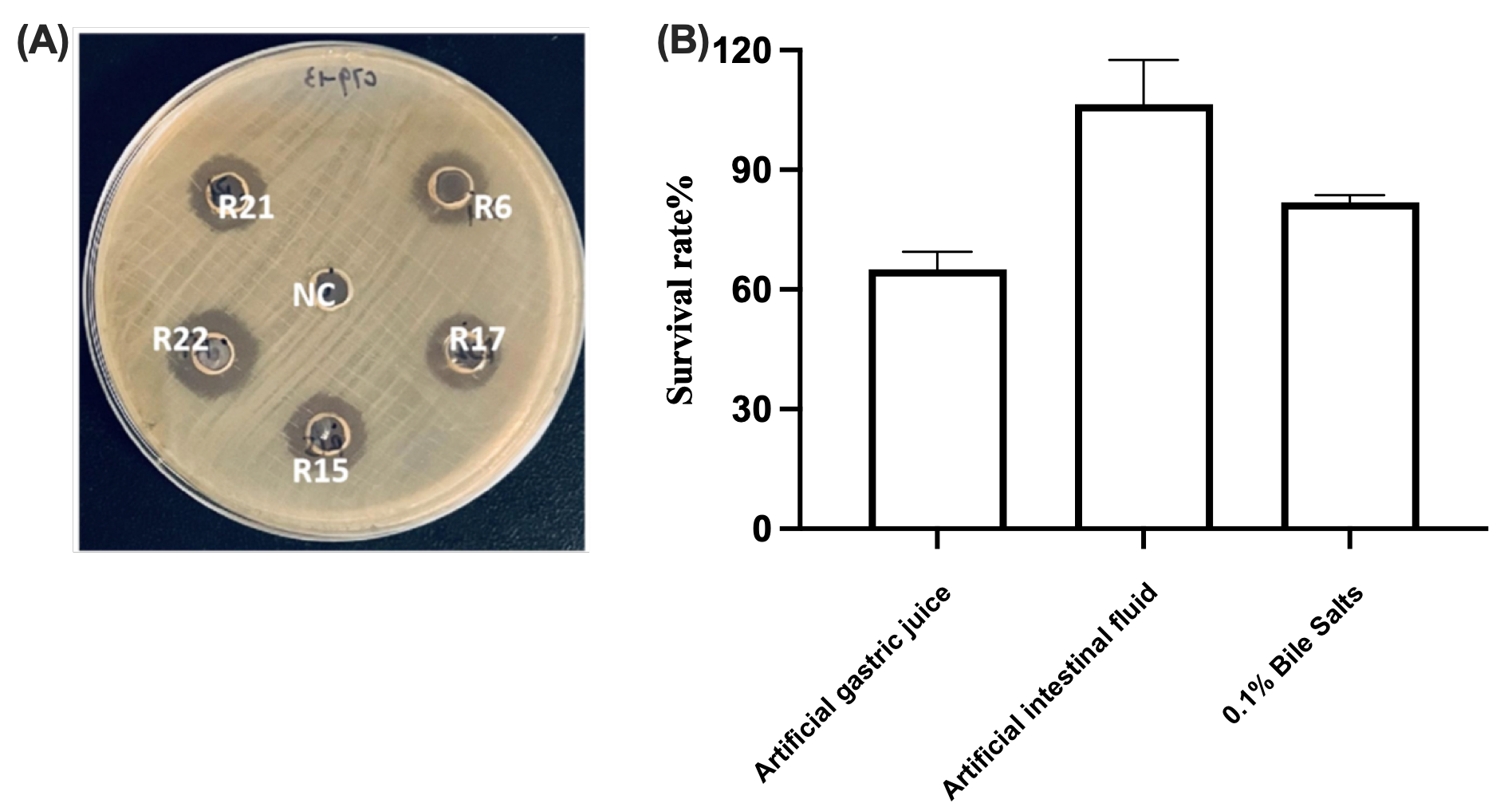

Supplement: Supplementary file 8 [file Image_8.tiff]
