# Supplementary material for: A chicken-origin Ligilactobacillus agilis R22 exerts probiotic features including growth-promotion and anti-Salmonella infection
Source: Front Microbiol. 2026 Jun 22;17:1862425. doi: 10.3389/fmicb.2026.1862425 (PMC13334848; doi:10.3389/fmicb.2026.1862425)
Supplement: Supplementary file 9 [file Supplementary_file_1.docx]

Supplementary Material

**Supplementary Figure S1.** *In vitro* screening of LAB strains. (A) Antimicrobial activity. (B) Survival rate of *L. agilis* R22 in simulated environments of digestive tracts

**Supplementary Figure S2.** General Genome Features and Taxonomic Identification of *L. agilis* R22. (A) Genome circle map. (B) System evolutionary tree. (C) ANI Analysis.

**Supplementary Figure S3.** Histogram of KEGG pathway classification statistics

**Supplementary Figure S4.** Relative abundance of the top 10 intestinal microbiota at the phylum and genus levels

**Supplementary Figure S5.** Wilcoxon rank-sum test box plot on genus level

**Supplementary Figure S6.** PLS-DA analysis (mix) in the samples of broiler chicken intestinal contents

**Supplementary Figure S7.** Key differential metabolic pathways and metabolites in the duodenum. (A) KEGG pathway enrichment analysis. (B) Box plots of metabolite distribution in each sample group

**Supplementary Figure S8.** Key differential metabolic pathways and metabolites in the cecum. (A) KEGG pathway enrichment analysis. (B) Box plots of metabolite distribution in each sample group

**Supplementary Table S1** Database annotation analysis

| Sample Name | NR No. | COG No. | GO No. | KEGG No. | Swiss-Prot No. | Pfam No. |
| --- | --- | --- | --- | --- | --- | --- |
| R22 | 2045 | 1518 | 1243 | 1399 | 1430 | 1617 |

**Supplementary Table S2** Probiotic feature gene annotation

| Gene | Function | Gene Numbers |
| --- | --- | --- |
| DNA and protein protection and repair | | |
| *folC* | folylpolyglutamate synthase/dihydrofolate synthase family protein | 1 |
| *luxS* | S-ribosylhomocysteine lyase | 2 |
| *recA* | recombinase RecA | 1 |
| *recN* | DNA repair protein RecN | 1 |
| pH stress resistance | | |
| *atpA* | F0F1 ATP synthase subunit alpha | 1 |
| *atpB* | F0F1 ATP synthase subunit A | 1 |
| *atpC* | F0F1 ATP synthase subunit epsilon | 1 |
| *atpD* | F0F1 ATP synthase subunit beta | 1 |
| *atpE* | F0F1 ATP synthase subunit C | 1 |
| *atpF* | F0F1 ATP synthase subunit B | 1 |
| *atpG* | F0F1 ATP synthase subunit gamma | 1 |
| *atpH* | ATP synthase F1 subunit delta | 1 |
| *nhaC* | Na^+^/H^+^ antiporter NhaC | 1 |
| *napA* | cation: proton antiporter | 2 |
| *rfbB* | dTDP-glucose 4,6-dehydratase | 1 |
| *gadC* | glutamate: GABA antiporter | 5 |
| *glnH* | glutamine ABC transporter substrate-binding and permease protein | 3 |
| *glnP* | aspartate/glutamate/glutamine transport system permease protein | 2 |
| *glnQ* | aspartate/glutamate/glutamine transport system ATP-binding protein | 2 |
| *pstS* | phosphate ABC transporter substrate-binding protein | 1 |
| *pstC* | phosphate ABC transporter permease subunit PstC | 1 |
| *guaA* | glutamine-hydrolyzing GMP synthase | 2 |
| *gene0918* | GTP pyrophosphokinase | 1 |
| Bile salt stress resistance | | |
| *ppaC* | manganese-dependent inorganic pyrophosphatase | 1 |
| *cbh* | choloylglycine hydrolase | 1 |
| *pyrG* | CTP synthase | 1 |
| *nagB* | glucosamine-6-phosphate deaminase | 1 |
| *cfa* | cyclopropane-fatty-acyl-phospholipid synthase family protein | 1 |
| Oxidative stress resistance | | |
| *trxA* | thioredoxin | 2 |
| *trxB* | thioredoxin reductase (NADPH) | 2 |
| *tpx* | thiol peroxidase | 1 |
| *msrA* | peptide-methionine (S)-S-oxide reductase MsrA | 1 |
| *msrB* | peptide-methionine (R)-S-oxide reductase MsrB | 1 |
| *msrC* | L-methionine (R)-S-oxide reductase | 1 |
| *clpC* | ATP-dependent Clp protease ATP-binding subunit | 2 |
| *mntH* | manganese transport protein | 2 |
| *gene0407* | metal ABC transporter solute-binding protein | 1 |
| *gene1392* | metal ABC transporter permease | 1 |
| *fnr* | NAD(P)/FAD-dependent oxidoreductase | 1 |
| *ndh* | NAD(P)/FAD-dependent oxidoreductase | 1 |
| *gene1858* | FAD-dependent oxidoreductase | 1 |
| *spxB* | pyruvate oxidase | 1 |
| *arsC* | arsenate reductase ArsC | 2 |
| *nrdH* | glutaredoxin-like protein NrdH | 1 |
| Heavy metal stress resistance | | |
| *copA* | lead, cadmium, zinc and mercury transporting ATPase | 3 |
| *corA* | magnesium transporter CorA family protein | 1 |
| *zurR* | Fur family transcriptional regulator | 1 |
| Temperature tolerance | | |
| *groEL* | chaperonin GroEL | 2 |
| *groES* | chaperonin GroES | 1 |
| *gene0375* | SepM family pheromone-processing serine protease | 1 |
| *clpB* | ATP-dependent chaperone ClpB | 1 |
| *clpC* | ATP-dependent Clp protease ATP-binding subunit | 2 |
| *clpE* | ATP-dependent Clp protease ATP-binding subunit | 1 |
| *clpX* | ATP-dependent Clp protease ATP-binding subunit ClpX | 1 |
| *clpP* | ATP-dependent Clp endopeptidase proteolytic subunit ClpP | 2 |
| *hrcA* | heat-inducible transcriptional repressor HrcA | 1 |
| *hslO* | Hsp33 family molecular chaperone HslO | 1 |
| *hslV* | HslU--HslV peptidase proteolytic subunit | 1 |
| *hslU* | ATP-dependent protease ATPase subunit HslU | 1 |
| *dnaK* | molecular chaperone DnaK | 1 |
| *dnaJ* | molecular chaperone DnaJ | 1 |
| *htpX* | heat shock protein HtpX | 1 |
| *ctsR* | transcriptional regulator of stress and heat shock response | 1 |
| *gene1559* | Heat shock protein Hsp20 | 1 |
| *cshB* | ATP-dependent RNA helicase CshB | 1 |
| *cspA* | cold-shock protein | 2 |
| Osmotic stress resistant | | |
| *proV* | glycine betaine/L-proline ABC transporter ATP-binding protein | 1 |
| Immunomodulation | | |
| *dltA* | D-alanine--poly(phosphoribitol) ligase subunit DltA | 1 |
| *dltB* | D-alanyl-lipoteichoic acid biosynthesis protein DltB | 1 |
| *dltC* | D-alanine--poly(phosphoribitol) ligase subunit DltC | 1 |
| *dltD* | D-alanyl-lipoteichoic acid biosynthesis protein DltD | 1 |
| Adhesion activity | | |
| *MucBP* | MucBP domain-containing protein | 3 |
| *fnbA* | fibronectin-binding protein A | 1 |
| *eno* | enolase | 1 |
| *pgi* | glucose-6-phosphate isomerase | 1 |
| *tuf* | elongation factor Tu | 1 |
| *dnaK* | molecular chaperone DnaK | 1 |
| *tpiA* | triose-phosphate isomerase | 1 |
| *glnA* | type I glutamate--ammonia ligase | 1 |
| *gapA* | type I glyceraldehyde-3-phosphate dehydrogenase | 1 |
| *pyk* | pyruvate kinase | 1 |
| *epsA* | protein tyrosine kinase modulator | 1 |
| *epsB* | protein-tyrosine kinase | 1 |
| *galA* | alpha-galactosidase | 2 |
| *lacZ* | beta-galactosidase | 1 |
| *srtA* | sortase A | 1 |
| *sadA* | trimeric autotransporter adhesin | 4 |
| *oppA* | oligopeptide transport system substrate-binding protein | 6 |
| Motility-related factors | | |
| *fliC* | flagellin | 2 |
| *fliG* | flagellar motor switch protein FliG | 1 |
| *motA* | flagellar motor protein MotA | 1 |
| *motB* | flagellar motor protein MotB | 1 |
| *mcp* | methyl-accepting chemotaxis protein | 5 |
| *cheA* | chemotaxis protein CheA | 1 |
| *cheB* | chemotaxis response regulator protein-glutamate methylesterase | 1 |
| *cheC* | chemotaxis protein CheC | 1 |
| *cheD* | chemotaxis protein CheD | 1 |
| *cheR* | protein-glutamate O-methyltransferase CheR | 1 |
| *cheY* | chemotaxis protein CheY | 1 |
| *cheW* | chemotaxis protein CheW | 2 |
| Production of lactic acid | | |
| *ldh* | L-lactate dehydrogenase | 2 |
| *ldhA* | D-2-hydroxyacid dehydrogenase | 2 |
| Food digestion | | |
| *mleA* | malolactic enzyme | 1 |
| *nplT* | glycoside hydrolase family 13 protein | 3 |
| *bglA* | glycoside hydrolase family 1 protein | 4 |
| *malL* | alpha-glucosidase | 1 |
| *malZ* | alpha-glucosidase | 1 |
| *dexB* | alpha-glucosidase | 1 |

**Supplementary Table S3** Number of metabolites after data preprocessing in the samples of *L. agilis* R22's CFS

| Ion Mode | Effective number of ion peaks | Identified Metabolites | Percentage, % |
| --- | --- | --- | --- |
| neg | 5288 | 1550 | 29.31 |
| pos | 7448 | 1514 | 20.33 |

**Supplementary Table S4** Synthase genes for partial nutrients of *L. agilis* R22

| Gene | Function | Gene Numbers |
| --- | --- | --- |
| Lysine | | |
| *lysA* | diaminopimelate decarboxylase | 1 |
| Threonine | | |
| *thrC* | threonine synthase | 1 |
| Isoleucine | | |
| *ilvE* | branched-chain amino acid aminotransferase | 1 |
| Tyrosine | | |
| *aspB* | aspartate aminotransferase | 1 |
| *hisC* | histidinol-phosphate aminotransferase | 1 |
| Alanine | | |
| *ala* | alanine dehydrogenase | 1 |
| Proline | | |
| *proC* | pyrroline-5-carboxylate reductase | 1 |
| Serine | | |
| *psp* | phosphoserine phosphatase | 1 |
| Succinate | | |
| *gabD* | succinate-semialdehyde dehydrogenase | 3 |
| *fccA* | fumarate reductase (cytochrome) | 1 |

**Supplementary Table S5** ANOSIM based on Bray‒Curtis distance

| ANOSIM | Statistic | *p* value |
| --- | --- | --- |
| Duodenum_Con vs. Duodenum_R22 | -0.029 | 0.599 |
| Cecum_Con vs. Cecum_R22 | 0.1 | 0.376 |

**Supplementary Table S6** Number of metabolites after data preprocessing in the samples of broiler chicken intestinal contents

| Ion Mode | Effective number of ion peaks | Identified Metabolites | Percentage, % |
| --- | --- | --- | --- |
| neg | 8914 | 1700 | 23.44 |
| pos | 7670 | 1798 | 19.07 |
| mix | 16584 | 3498 | 21.09 |

**Supplementary Table S7** Concentrations of SCFAs (ng/mg) in the samples of broiler chicken intestinal contents

| Items | Acetic acid | Propionic acid | Isobutyric acid | Butyric acid | Isobutyric acid | Valeric acid | Isohexanoic acid | Hexanoic acid |
| --- | --- | --- | --- | --- | --- | --- | --- | --- |
| Con | 1208.55±295.99 | 232.29±90.42 | 54.31±18.41 | 474.29±205.98 | 36.20±15.26 | 59.04±14.35 | 0.85±0.31 | 1.99±0.60 |
| R22 | 1233.77±365.15 | 191.20±87.75 | 40.81±20.94 | 440.10±231.22 | 23.01±13.85 | 57.93±19.58 | 0.80±0.77 | 1.94±0.68 |
| *p* value | 0.8815 | 0.3719 | 0.1927 | 0.7594 | 0.0917 | 0.8989 | 0.8923 | 0.8715 |
